# Targeting TRAF3IP2, Compared to Rab27, is More Effective in Suppressing the Development and Metastasis of Breast Cancer

**DOI:** 10.1038/s41598-020-64781-z

**Published:** 2020-06-01

**Authors:** Eckhard U. Alt, Philipp M. Wörner, Andreas Pfnür, Joana E. Ochoa, Deborah J. Schächtele, Zahra Barabadi, Lea M. Lang, Sudesh Srivastav, Matthew E. Burow, Bysani Chandrasekar, Reza Izadpanah

**Affiliations:** 1 Applied Stem Cell Laboratory, Department of Medicine, Heart and Vascular Institute, Tulane University School of Medicine, New Orleans, Louisiana USA; 2 Department of Surgery, Tulane University School of Medicine, New Orleans, Louisiana USA; 3 Department of Global Biostatistics and Data Science, Tulane University School of Public Health & Tropical Medicine, New Orleans, Louisiana USA; 4 Department of Medicine, Section of Hematology & Medical Oncology, Tulane University School of Medicine, New Orleans, Louisiana USA; 5 Department of Medicine, University of Missouri School of Medicine and Harry S. Truman Veterans Memorial Hospital, Columbia, Missouri USA

**Keywords:** Breast cancer, Cancer models, Mesenchymal stem cells

## Abstract

Here we investigated the roles of Rab27a, a player in exosome release, and TRAF3IP2, an inflammatory mediator, in development and metastasis of breast cancer (BC) *in vivo*. Knockdown (KD) of Rab27a (MDA_KDRab27a_) or TRAF3IP2 (MDA_KDTRAF3IP2_) in triple negative MDA-MB231 cells reduced tumor growth by 70–97% compared to wild-type tumors (MDA_w_). While metastasis was detected in MDA_w_-injected animals, none was detected in MDA_KDRab27a_- or MDA_KDTRAF3IP2_-injected animals. Interestingly, micrometastasis was detected only in the MDA_KDRab27a_-injected group. In addition to inhibiting tumor growth and metastasis, silencing TRAF3IP2 disrupted inter-cellular inflammatory mediator-mediated communication with mesenchymal stem cells (MSCs) injected into contralateral mammary gland, evidenced by the lack of tumor growth at MSC-injected site. Of translational significance, treatment of pre-formed MDA_w_-tumors with a lentiviral-TRAF3IP2-shRNA not only regressed their size, but also prevented metastasis. These results demonstrate that while silencing Rab27a and TRAF3IP2 each inhibited tumor growth and metastasis, silencing TRAF3IP2 is more effective; targeting TRAF3IP2 inhibited tumor formation, regressed preformed tumors, and prevented both macro- and micrometastasis. Silencing TRAF3IP2 also blocked interaction between tumor cells and MSCs injected into the contralateral gland, as evidenced by the lack of tumor formation on MSCs injected site. These results identify TRAF3IP2 as a novel therapeutic target in BC.

## Introduction

Currently, about one in eight women (~12.4%) in United States (US) develop breast cancer (BC). It is estimated that 268,600 new cases of invasive and 62,930 of non-invasive BC will be diagnosed in 2019 in US alone, resulting in an estimated 41,760 deaths^1^. In addition to genetic and epigenetic alterations, the cellular and acellular compartments of the tumor microenvironment (TME) contribute to tumor growth and dissemination^2^.

The cellular fraction of the TME includes myofibroblasts, infiltrating fibroblasts, endothelial cells, stromal/stem cells and immune cells^3^. The acellular fraction of the TME comprises of exosomes, components of the extracellular matrix and soluble mediators such as cytokines and growth factors^3^. The interactions between cellular and acellular fractions of the TME form the basis of pro-tumorigenic signaling, promoting further tumor growth and metastasis^4^. We previously reported that these interactions alter gene expression in the cellular fraction of the TME^5^. We also reported that exposure of naïve mesenchymal stem cells (MSCs) to the acellular compartment of the TME results in their transformation into tumor-forming cells, confirming a critical role for the TME in tumor growth^6^.

The acellular fraction of the TME comprises of exosomes^7^. Exosomes are small vesicles (40–100 nm in diameter) released into extracellular space and play a role in cell-cell communication^8^. It has been shown that exosomes of non-malignant cells, such as MSCs, play a crucial role in tumor progression and increased tumor cell migration^9–11^. MSCs on the other hand can be transformed by exosomes of breast cancer cells in tumor-like cells, with the ability of tumor growth *in vivo*^6^. One key characteristic of exosomes is that their origin lies within the endosome. The early endosome matures into a multivesicular endosome (MVE, also called multivesicular body) and accumulates hundreds of intra-luminal vesicles due to inward budding of the endosomal membrane^12^. The Rab GTPase protein family and Rab effector molecules, specifically Rab27a (Rab effector molecule regulating exocytosis of exosomes), play a central role in promoting fusion of MVE to the cell membrane and the release of exosomes into the extracellular space^13^.

Cytokines, the soluble mediators of the acellular fraction of the TME, contribute to tumor progression by promoting angiogenesis and amplifying inflammation^14,15^. The dimeric nuclear transcription factor NF-κB is well known to transcriptionally upregulate several pro-tumorigenic mediators^16^, including cytokines. Under basal conditions, the NF-κB  dimer is localized in cytoplasm due to its binding to an inhibitory subunit called IκB. The IKK signalosome induces phosphorylation of IκB, resulting in its dissociation and degradation in cytoplasm. The free NF-κB then translocates to the nucleus^17^. Because of their crucial role in the transcriptional regulation of several pro-tumorigenic and proinflammatory mediators, both IKK and NF-κB have been extensively investigated as potential targets in BC growth and metastasis, but with discouraging results. The adaptor molecule TRAF3IP2 (TRAF3 Interacting Protein 2) is an upstream regulator of NF-κB, AP-1 and stress-activated kinases, and its sustained activation contributes to tumor progression. The activation of TRAF3IP2 is mainly associated with IL-17 signaling. IL-17 mediates cellular immune responses and a dominant “signature” cytokine of TH-17 cells, which upregulates cytokines, neutrophil-mobilizing chemokines, and tissue-degrading matrix metalloproteases^18^.

Until today, it is not clear whether the insoluble or soluble fraction of the TME has the bigger impact on tumor development and progression. To further investigate this question, we focused on Rab27a as a representative of the insoluble (exosomes) fraction and TRAF3IP2 as the representative of the soluble (proinflammatory mediators) fraction of the TME. Since Rab27a and TRAF3IP2 contribute to exosome release and inflammation, respectively, we hypothesized that targeting Rab27a or TRAF3IP2 will reduce inflammation, tumor formation, growth and metastasis of BC in a preclinical breast xenograft model.

## Methods

### Cell culture

#### MDA-MB231 cancer cell line and 184A1 normal human breast cell line

The triple negative human breast cancer cell line MDA-MB231 (Cat# AKR-201) was purchased from Cell Biolabs, Inc (San Diego, CA), and was authenticated by the vendor. This malignant cell line was originally obtained in 1973 from a patient at the M. D. Anderson Cancer Center in Houston, TX. The cells were positive for mutation in the proto-oncogene KRAS (heterozygous DNA change: c.38 G > A; correlates to protein sequence p.G13D). The cells were pathogen-free (negative for HIV, HepB, HPV, EBV, and CMV by PCR). They have epithelial-like morphology and appear as spindle shaped cells. During the course of the experiments, we routinely verified their morphology under phase contrast microscope. The cells were able to grow on agarose (anchorage-independence), an indication of transformation and tumorigenicity, and display a relatively high colony forming efficiency. We monitored their tumorigenic potential every 6 months by intramammary injection (5 × 10^5^ cells in Matrigel). The cells were cultured in MEM-Alpha Growth Medium (Cat#15-012-CV, CellGro, Manassas, VA) containing 10% FBS (Cat#511550, Atlanta Biologicals, Lawrenceville, GA), 1% Penicillin/Streptomycin (Cat#30-002-CI, Cellgro) and 1% L-Glutamine (Cat#25-005-CI, Cellgro). The culture medium was free of endotoxin as analyzed by Limulus Amoebocyte Lysate Assay (Pierce LAL Chromogenic Endotoxin Quantitation Kit, Catalog# 88282, Thermo Fisher Scientific, Waltham, MA).

184A1 cells, established from normal mammary tissue (kindly provided by Dr. Martha Stampfer, Lawrence Berkeley Laboratory, Berkeley, CA), was cultured in DMEM/F12 Medium supplemented with 5% Horse Serum (Cat#H1270, Sigma-Aldrich, St. Louis, MO), 100 U/mL Penicillin, 100 μg/mL Streptomycin, 20 ng/mL hEGF (Cat# AF-100-15, Peprotech, Rocky Hill, NJ), 10 μg/mL Insulin (Cat#I0516, Sigma-Aldrich) and 500 ng/mL Hydrocortisone (Cat#H0888, Sigma-Aldrich), at 37 °C in an atmosphere containing 5% CO_2_.

#### Human adipose tissue-derived mesenchymal stem cells (MSCs)

Following the guidelines of International Conference on Harmonization (ICH) E-6 Good Clinical Practice and approval by the Tulane University Institutional Review Board (IRB#140571) and informed written consent, adipose tissue specimens were collected from healthy subjects undergoing cosmetic surgery. The MSCs were isolated as previously described^19^. As recommended by the International Society of Cellular Therapy (ISCT), MSCs were characterized for the surface expression of CD4, CD11b, CD34, CD45, CD44, CD73, CD90, CD105, and HLA-DR by flow cytometry using a Beckman-Coulter Epics FC500^19–21^. The MSCs were negative for CD4, CD11b, CD34, CD45 and HLA-DR, but positive for CD44, CD73, CD90, and CD105. The multilineage potential of MSCs was examined by adipogenic, osteogenic, and chondrogenic differentiation assays according to established methods^22,23^.

### Rab27a and TRAF3IP2 mRNA expression in MDA-MB231, 184A1 and MSCs in single and cocultures

Wildtype MDA-MB231 (MDA_w_) cells were cocultured with either 184A1 or MSCs in a Boyden Chamber System, separated by a membrane with 1μm-pore size that allows for communication without a direct contact. After 48 hours, total cellular RNA was extracted, reverse transcribed, and analyzed for Rab27a (Forward: 5′-GCCACTGGCAGAGGCCAG-3′; Reverse: 5′-GAGTGCTATGGCTTCCTCCT-3′) and TRAF3IP2 (Forward: 5′-AACAAGCAATTTGCCAGAAG-3′; Reverse: 5′-TGTTTGTATTTGGGGCTGAT-3′) by Real-time PCR. GAPDH (Forward: 5′-GGAAGGACTCATGACCACAG-3′; Reverse: 5′-TTGGCAGGTTTTTCTAGACG-3′) served as a housekeeping gene.

### Knockdown of Rab27a or TRAF3IP2 in MDA-MB231 cells and MSCs

MDA-MB231 cells and MSCs were transduced with lentiviral shRNA (MOI = 1) against Rab27a (Cat#sc-41834-V, Santa Cruz Biotechnology, Inc., Dallas, TX) or TRAF3IP2 (TRCN000015477, Sigma-Aldrich). Polybrene (5 µg/ml; Cat#sc-134220, Santa Cruz Biotechnology, Inc.) was used to increase transduction efficiency. Puromycin Dihydrochloride (Cat#sc-108071, Santa Cruz Biotechnology, Inc) was used to select Rab27a or TRAF3IP2 silenced cells. Knockdown of Rab27a (MDA_KDRab27a_) and TRAF3IP2 (MDA_KDTRAF3IP2_) was confirmed by Western Blot.

### Conditioned medium (CM)

The CM is the supernatant of a cell culture medium without the FBS, and was collected according to a published method^24,25^. Briefly, MDA-MB231 cells were grown to 85% confluency in 10 cm dishes (Nalgene, Nunc, Rochester, NY), washed twice with Phosphate-Buffered Saline (PBS; CellGro), and incubated at 37° for 72 hours in serum-free MEM-Alpha Medium containing the supplements. The culture supernatants were then collected, centrifuged (300 × g for 10 minutes followed by 1,200 × g for 10 minutes), filter sterilized through a 0.2μm filter, and stored at −20 °C until use.

### Exosome enrichment

To concentrate exosomes from the MDA-MB231 cell-derived CM, we used a series of ultracentrifugation steps as previously described^7^. The CM was spun at 115,000 × *g* for 1 hour at 4 °C in an SW41Ti Ultracentrifuge (Beckman Coulter, Fullerton, CA). After discarding 90% of the supernatant, 3.6 ml of PBS was added and layered onto a 30% sucrose/D_2_O density cushion and spun at 115,000 × *g* for 1 hour at 4 °C. The supernatant (700 µl) was collected and spun again at 115,000 × *g* for 1 hour at 4 °C. The resulting pellet was re-suspended in PBS and centrifuged at 115,000 × *g* for 1 hour at 4 °C. Following one last wash, the pellet was re-suspended in PBS and stored at −80 °C. The concentration of exosomes was determined by quantifying the protein content using NanoDrop 2000 (Thermo Fisher Scientific, Waltham, MA).

### RT^2^ profiler PCR array

A RT² Profiler PCR Array Human Common Cytokines (Prod.No.: PAHS-021A-2, Qiagen, Valencia, CA) was used to analyze gene expression in MDA_w_ and MDA_KDTRAF3IP2_ cells. RNA was extracted using RNeasy Mini Kit (Cat#74104, Qiagen). cDNA was synthesized using RT^2^ First Strand Kit (Cat#330401, Qiagen).

### Western blotting

M-PER Mammalian Protein Extraction Reagent (Cat#78503, Thermo Fisher Scientific, Waltham, MA) together with Proteinase Inhibitor Cocktail (Cat#P8340, Sigma Aldrich, St. Louis, MO) were used to extract proteins from MDA_w_, MDA_KDRab27a_ and MDA_KDTRAF3IP2_ cells or from conditioned media of MDA_w_ (EXO_MDAw_) or MDA_KDRab27a_ (EXO_MDAKDRab27a_) cells. After gel electrophoresis of equal amounts of protein using 12% Precise Tris-Glycine Gels (Cat#0025267, Thermo Fisher Scientific), Laemmli Sample Buffer (Cat#161-0747, BioRad Laboratories, Hercules, CA) and BenchMark Pre-Stained Protein Ladder (Cat#10748-010, Invitrogen, Carlsbad, CA) the proteins were electroblotted and the following primary antibodies were used: GAPDH (0.0002 mg/ml; Cat#ab9485, Abcam, Cambridge, MA), Rab27a (0.01 mg/ml; Cat#sc-22756, Santa Cruz Biotechnology, Inc.), TRAF3IP2 (0.01 mg/ml; Cat#WH0010758M1-100UG, Sigma-Aldrich), CD9 (0.01 mg/ml; Cat#MA1-19002, Thermo Fisher Scientific), or MHCII (0.01 mg/ml; Cat#MA1-19143, Thermo Fisher Scientific). Goat Anti-Rabbit IgG-HRP (Cat#sc-2004, Santa Cruz Biotechnology, Inc.) or Donkey Anti-Mouse IgG-HRP (Cat#sc-2318, Santa Cruz Biotechnology, Inc.) served as secondary antibodies.

### Ultrastructural analysis by electron microscopy

MDA_w_, MDA_KDRab27a_, and MDA_KDTRAF3IP2_ cells were cultured in regular medium, washed in PBS, and fixed in 2.5% Glutaraldehyde (Cat#G5882, Sigma-Aldrich) for 30 minutes. The secondary fixation step consisted of 4% Osmium Tetroxide (Cat#75632, Sigma-Aldrich), washed in distilled water, and dehydrated in graded alcohol. Critical point dry coating with gold alloy and imaging were performed with a Hitachi S-4800 Field Emission Scanning Electron Microscope (Hitachi America, Tarrytown, NY).

### Human tumor invasion/metastasis primer library

Human Tumor Invasion/Metastasis Primer Library (Real Time Primers, Elkins Park, PA) consisted of 88 primer sets directed against tumor invasion/metastasis genes. GAPDH was used as a housekeeping gene. The extraction of RNA and construction of cDNA were performed using RNeasy Mini Kit (Cat#74104, Qiagen), RT^2^ First Strand Kit (Cat#330401, Qiagen) and High-Capacity cDNA Reverse Transcription Kit (Cat#4374966, Applied Biosystems, Forster City, CA). PCR was performed in triplicates using FastStart Universal SYBR Green Master (Cat#0491385001, Roche Diagnostics Cooperation, Indianapolis, IN) according to the manufacturer’s protocol. Relative gene expression was measured by CT and fold change calculated as previously described^26^.

### Cell cycle analysis

For analysis, cells were counted after 48, 96 and 120 hours. At each time point, doubling time and population doublings were calculated using the previously described equation: log_10_ [N/N_0_] × 3.33^27^ where N represents total number of cells at each time point and N_0_ the number of seeded cells.

### *In vivo* experiments

All animal protocols were approved by the Institutional Animal Care and Use Committee at the Tulane University School of Medicine in New Orleans, LA, and conformed to the Guide for the Care and Use of Laboratory Animals, published by the National Institutes of Health (DRR/National Institutes of Health, 1996). Female 6–8-week-old immunodeficient NIH-III nude mice (hereafter referred to as nude mice) were purchased from Charles River Laboratories, Inc. (Wilmington, MA), and maintained in a 12-hour light/dark cycle barrier facility, with food and water available *ad libitum*.

### Experimental series I: Intramammary administration of MDA_w_, MDA_KDRab27a,_ and MDA_KDTRAF3IP2_ cells

MDA_w_, MDA_KDRab27a_ or MDA_KDTRAF3IP2_ cells suspended in Matrigel (5 × 10^5^ cells in 100 μl, Cat#354234, Corning, NY, USA) were injected subcutaneously into the fourth left mammary gland of nude mice (MDA_w_: n = 3, MDA_KDRab27a_ or MDA_KDTRAF3IP2_: n = 6, Fig. 4A–C: Schematic experimental design). After 8 weeks, nude mice injected with MDA_w_ were euthanized due to larger tumors. After 15 weeks, one half of the population of nude mice injected with MDA_KDRab27a_ or MDA_KDTRAF3IP2_ cells were euthanized and residual tumors collected for further analysis. The other half was used for evaluating lifespan at week 52. Two groups of animals (n = 3) injected with PBS or Matrigel on both sides served as controls. All animals were monitored for 52 weeks (Supplementary Figure S1A,B). After euthanasia, tumors were weighed and their volumes calculated [1/2(length × width^2^)] as previously described^28^.

### Experimental series II: Administration of MDA_w_, MDA_KDRab27a_ or MDA_KDTRAF3IP2_ cells and naïve MSCs into contralateral mammary glands

To evaluate the impact of crosstalk between naïve MSCs and MDA_w_, MDA_KDRab27a_ or MDA_KDTRAF3IP2_ cells (5×10^5^ cells in 100 μl Matrigel) on tumor growth, nude mice were injected with MDA_w_ (n = 3), MDA_KDRab27a_ or MDA_KDTRAF3IP2_ (n = 6 each) into the fourth left mammary gland. Naïve MSCs (5 × 10^5^ cells in 100 μl Matrigel) were injected into the contralateral gland (schematic experimental design Fig. 5A–C). After 8 weeks, mice injected with MDA_w_ and MSCs were euthanized due to larger tumors. In accordance with the previous experiment, half of mice injected with MDA_KDRab27a_/MSCs as well as MDA_KDTRAF3IP2_/MSCs were euthanized after 15 weeks and tumors collected for further analysis. Three animals each were kept for survival studies for up to 52 weeks (endpoint of the experiment). Animals injected with PBS, Matrigel or naïve MSCs served as controls (n = 3 mice/group; Supplementary Figure S1A–C).

### Experimental series III: Administration of MDA_w_ cells into nude mice with subsequent treatment with lentivirus expressing TRAF3IP2 shRNA

In this translationally important strategy, tumors were induced at first by injecting MDA_w_ cells expressing the luciferase gene (5×10^5^ cells/100 μl Matrigel) into fourth left mammary gland of nude mice (n = 6). After confirming tumor growth at 14 days, the mice were divided into two groups (n = 3): Group 1 was injected with lentiviral TRAF3IP2 shRNA (MOI = 1) on to the tumor surface, Group 2 was injected with a GFP-labeled lentiviral vector containing scrambled shRNA (control) twice a week. Animals were euthanized after 8 weeks and tumors collected for further analysis. Prior to euthanasia, luciferase expression was analyzed by IVIS Lumina XRMS *In Vivo* Imaging System (PerkinElmer, Waltham, MA).

### Histology

Tumors sections (4 μM-thick) were stained for H&E, Cytokeratin AE1/AE3 (Cat#M3515, Agilent, CA), IL8 (Cat#ab84995, Abcam, MA), Ki67 (Cat#Ab16667, Abcam) or Caspase-3 (Cat#Ab32351, Abcam) according to the manufacturer’s protocol, and evaluated using a Leica Microscope.

### Detection of micrometastasis by PCR

Micrometastasis was evaluated by PCR using genomic DNA (QIAamp DNA Mini Kit, Cat#51304, Qiagen) isolated from brain, kidney, lung, liver, spleen and bone from MDA_w_-, MDA_KDRab27a_-_,_ and MDA_KDTRAF3IP2_-injected nude mice using primers that specifically detect α-satellite DNA sequence of the centromere region of human chromosome 17^29^ (Forward, 5′-GGGATAATTTCAGCTGACTAAACAG-3′ and reverse, 5′-TTCCGTTTAGTTAGGTGCAGTTATC-3′; IDT, Coralville, IA). Genomic DNA from healthy mouse tissue served as a control.

### Statistics

All data relating to study specific was summarized using descriptive statistics such as mean, standard deviation and standard error. Estimates of mean difference and their 95% confidence intervals were calculated. The analysis of variance method was used to compare the mean differences. Where meaningful, the results were presented graphically. The study hypotheses were tested at 5% level of significance throughout the analysis. (*P ≤ 0.05; **P ≤ 0.01; ***P ≤ 0.001; ****P ≤ 0.0001).

## Results

### *In vitro* studies

#### MDA-MB231 cells express high levels of Rab27a and TRAF3IP2

**Figure 1:**
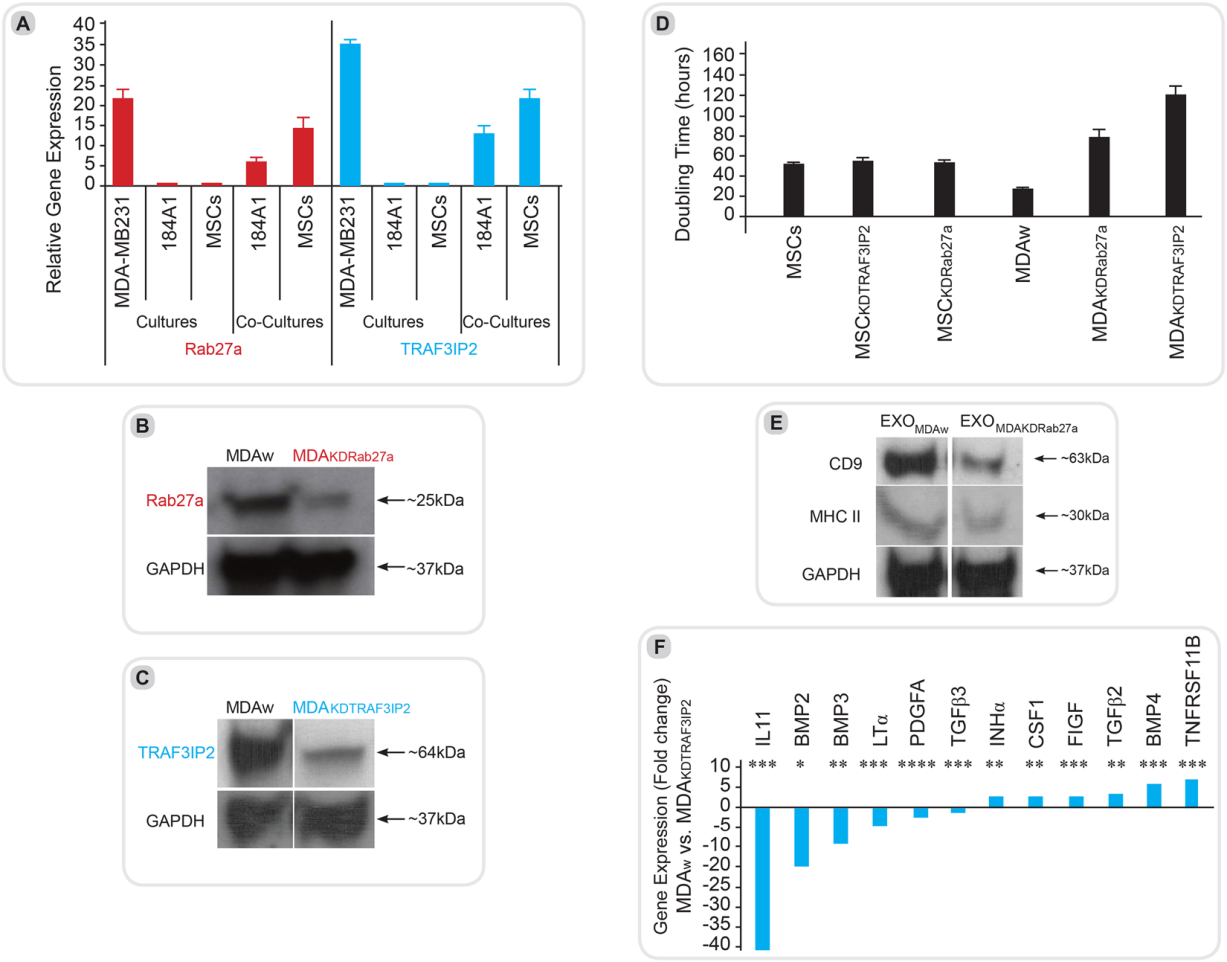
Expression levels of Rab27a and TRAF3IP2 in malignant and non-malignant breast cells. The graph shows the gene expression of TRAF3IP2 and Rab27a in cultures of MDA-MB231 (MDA_w_), 184A1 and MSCs including the standard deviation. The expression of both genes, TRAF3IP2 and Rab27a, are significantly higher in MDA_w_ than in the non-malignant cells lines such as 184A1 and MSCs. After coculturing 184A1 or MSCs for 48 hours with MDA_w_, the expression of TRAF3IP2 and Rab27a in both 184A1 and MSCs increased significantly. (**B,C**) Western blots of MDA-MB231 (MDA_w_), MDA_KDRab27a_ and MDA_KDTRAF3IP2_ cells stained with antibodies for Rab27a, TRAF3IP2 or GAPDH. GAPDH served as a housekeeping marker. A significant decrease in Rab27a and TRAF3IP2 protein levels is seen in cells after transduction with shRNA for Rab27a or TRAF3IP2 (MDA_KDRab27a_ and MDA_KDTRAF3IP2_). Displayed are cropped blots, for full blots see Supplementary Data. **(D**) Effect of Silencing Rab27a and TRAF3IP2 in malignant and non-malignant breast cell proliferation shows MDA-MB231 and MSCs doubling time before and after transduction with Rab27a and TRAF3IP2 shRNA. The reduction of Rab27a as well as TRAF3IP2 expression in MDA-MB231 cells result in comparison to MDA_w_ in a decreased cell proliferation and therefore longer doubling time. No effects of Rab27a or TRAF3IP2 knockdown were seen in MSCs replication capacity. (**E**) Exosome content in knock down cells assessed using western blots analysis of condition media of MDA_w_ and MDA_KDRab27a_ using CD9 and MHCII as marker for exosomes. Data show that the expression of CD9 as well as MHCII decrease in EXO_MDAKDRab27a_ in comparison to EXO_MDAw_. Displayed are cropped blots, for full blots see Supplementary Data. **(F**) Effect of TRAF3IP2 silencing on the expression of cytokines. The group plot displays the result of a RT² Profiler PCR Array Human Common Cytokines performed with the mRNA of MDA_w_ and MDA_KDTRAF3IP2_. MDA_w_ was used as the baseline. All shown results are of significance (Legend: *p < 0.05; **p < 0.01; ***p < 0.001; ****p < 0.0001). Higher gene expression levels in comparison of MDA_KDTRAF3IP2_ to MDA_w_ were found in TNFRSF11B, TGFβ2, BMP4 as well as CSF1, FIGF and INHα. A downregulation in gene expression was detected in BMP2, BMP3, IL11, LTα, PDGFA and TFGβ3.

The expression levels of both Rab27a and TRAF3IP2 were significantly higher in MDA-MB231 cells compared to 184A1, a normal BC cell line, and naïve MSCs (Fig. 1A). Interestingly, when cocultured with MDA-MB231 cells, the expression levels of Rab27a and TRAF3IP2 were markedly elevated in both 184A1 cells and MSCs (Fig. 1A), strongly suggesting that soluble mediators from MDA-MB231 cells affect gene expression in non-malignant epithelial and stromal cell populations in a paracrine manner.

#### Silencing Rab27a or TRAF3IP2 alters characteristics of MDA-MB231 cells

Silencing Rab27a by a lentiviral Rab27a shRNA (MDA_KDRab27a_) suppressed Rab27a expression in MDA-MB231 cells (Fig. 1B). Similarly, lentiviral transduction of TRAF3IP2 shRNA (MDA_KDTRAF3IP2_) suppressed its expression (Fig. 1C). Interestingly, silencing Rab27a and TRAF3IP2 each significantly increased population doubling times of MDA-MB231 cells, but not that of Rab27a- or TRAF3IP2-silenced MSCs (Fig. 1D). Further, western blot analysis demonstrated a marked reduction in exosome-specific markers *CD9* and *MHCII*^30^ in MDA_KDRab27a_-derived culture supernatants, indicating that Rab27a knockdown blunts exosome release (Fig. 1E).

#### Silencing TRAF3IP2 differentially regulates cytokine expression

In comparison to Rab27a, which plays a critical role in exosome release, TRAF3IP2 contributes to inflammation by regulating multiple proinflammatory and pro-tumorigenic mediators. Transcriptomic analysis of MDA_KDTRAF3IP2_ cells by RT² Profiler PCR Array identified a cluster of 12 pro-inflammatory genes that was differentially expressed (>2-fold) in MDA_KDTRAF3IP2_ cells compared to MDA_w_ cells (Fig. 1F). For example, the expression of *IL11* (Interleukin 11), a gene involved in promoting migration and invasion in BC cells, is reduced in MDA_KDTRAF3IP2_ cells^31^. The expression of *BMP2* (Bone morphogenetic protein 2) and *BMP3* were decreased, while *BMP4* expression was increased. In addition, silencing TRAF3IP2 differentially regulated the expression of *TGFβ* (Transforming Growth Factor β) superfamily members *TGFβ2* and *TGFβ3;* while the expression of *TGFβ2* was increased, expression of *TGFβ3* was decreased. The expression of *TNFRSF11B* (Tumor Necrosis Factor Receptor Superfamily Member 11b) was also increased. Silencing TRAF3IP2 also reduced *LTα* (Lymphotoxin Alpha) and *PDGFA* (Platelet Derived Growth Factor Subunit A) expression in MDA-MB231 cells. However, silencing TRAF3IP2 resulted in upregulation of *INHα* (Inhibin Subunit Alpha), *CSF1* (Colony Stimulating Factor 1) and *FIGF* (C-Fos Induced Growth Factor or *VEGF-D*: Vascular Endothelial Growth Factor D) expression.

#### Silencing Rab27a or TRAF3IP2 alters gene expression in MDA-MB231 cells

**Figure 2:**
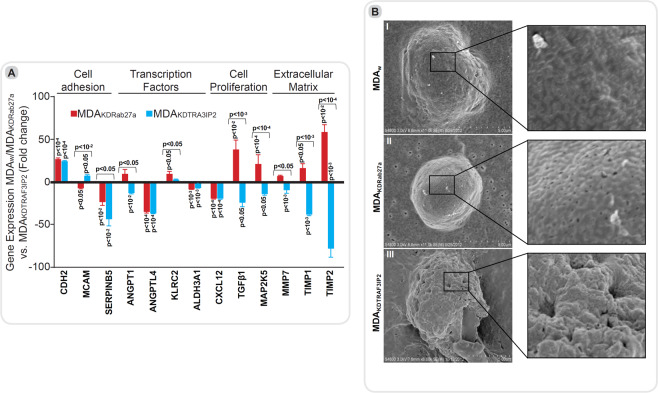
(**A**) Changes in gene expression in^levels of^ MDA-MB231 cells after silencing Rab27a and TRAF3IP2. Using PCR, the expression of selected genes in^expressions of^ MDA_w_, MDA_KDRab27a_ and MDA_KDTRAF3IP2_ were compared. Gene expression of MDA_w_ was set to baseline in the graphs. Then the genes were grouped based on function into cell adhesion, transcription factors, cell growth and proliferation, and extracellular matrix. The graphs are representatives of triplicate experiments. The analysis of variance method was used for the statistical analysis of each gene. (**B**) Electron microscopy. The morphology of MDA_w_ (B.I), MDA_KDRab27a_ (B.II), and MDA_KDTRAF3IP2_ cells (B.III) is shown. The cells were stained and viewed with a Hitachi S-4800 Field Emission Scanning Electron Microscope. The scale bars are 5 μm. By comparing the wildtype (MDA_w_) to MDA_KDRab27a_ and MDA_KDTRAF3IP2_ no significant difference in size (B.I-B.III) was seen. The surface of MDA_KDRab27a_ and MDA_KDTRAF3IP2_ seem to be in a very inhomogeneous state. Higher magnification shows porous cell surface in MDA_KDRab27a_ cells. MDA_KDTRAF3IP2_ exhibited a rough and shrunk surface compared to MDA_w_.

Transcriptomic analysis of MDA_KDRab27a_ or MDA_KDTRAF3IP2_ cells showed differential expression of genes involved in cell adhesion, transcription factors, cell growth, cell proliferation, and extracellular matrix proteins compared to MDA_w_ cells (Fig. 2A). For example, silencing Rab27a or TRAF3IP2 increased *CDH2* (N-Cadherin) expression compared to MDA_w_ cells. Furthermore, expression of *SERPINB5* (Serpin Family B Member 5) was decreased in both MDA_KDTRAF3IP2_ and MDA_KDRab27a_ cells. Interestingly, while the expression of MCAM (Melanoma Cell Adhesion Molecule) was increased in MDA_KDTRAF3IP2_ cells, its expression was decreased in MDA_KDRab27a_ cells. Among the genes regulating transcription factors, expression of both *ANGPTL4* (Angiopoietin Like 4) and *ALDH3A1* (Aldehyde Dehydrogenase 3 Family Member A1) were significantly decreased in MDA_KDTRAF3IP2_ and MDA_KDRab27a_ cells. The expression of *CXCL12*, a gene involved in cell proliferation, is significantly downregulated in both MDA_KDTRAF3IP2_ and MDA_KDRab27a_ cells. Interestingly, the data also show that the expression of extracellular matrix proteins were only downregulated in MDA_KDTRAF3IP2_ cells (Fig. 2A, detailed statistical analysis is provided in Supplementary Table 1).

In addition, electron microscopy showed significant morphological changes in both MDA_KDRab27a_ and MDA_KDTRAF3IP2_ cells (Fig. 2B); while MDA_KDRab27a_ showed porous cell surfaces (Fig. 2BII), MDA_KDTRAF3IP2_ cells exhibited rough and shrunken surfaces (Fig. 2BIII) compared to MDA_w_ (Fig. 2BI). However, the cell size was not markedly altered in the silenced cells (Fig. 2BI–III).

#### *In vivo* studies in a breast xenograft model

**Figure 3:**
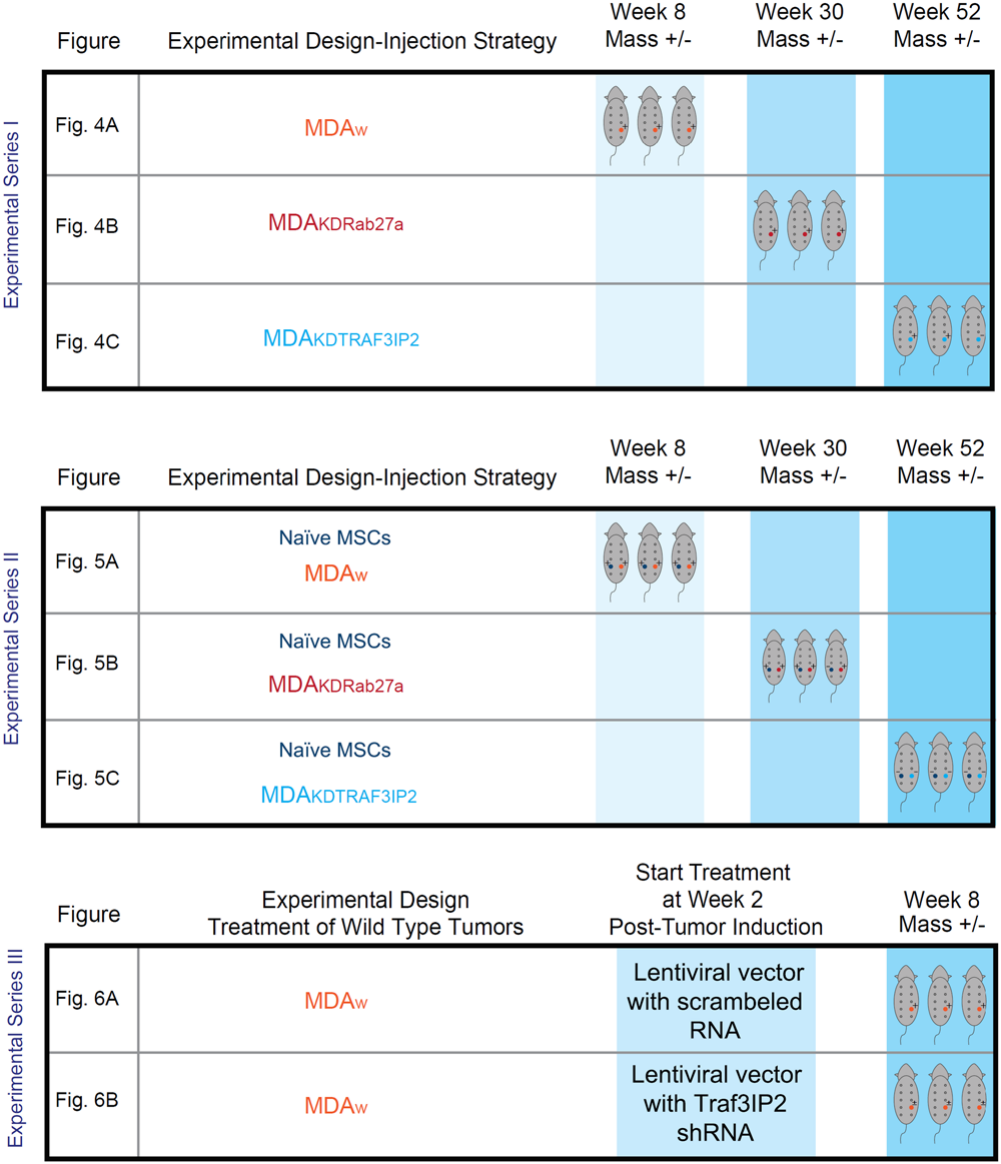
Experimental Design-Injection Strategy. The schematic summarizing the experimental design of experimental series I, II, and III. The column on the left indicates the data of experiments I-III summarized in Figs. 4–6. The next column describes the cells injected subcutaneously in the mice displayed in the columns to the right. The color pattern of the middle and the right columns correlate. A plus sign (+) in the right column symbolizes growth of a tumor or in case of injected MSCs a tumor-like mass. A minus sign (−) symbolizes no growth. A plus/minus sign (±) in the bottom row (Fig. 6) symbolizes a significantly reduced tumor growth in comparison to MDA_w_.

The overview of the *in vivo* experiments is displayed in Fig. 3.

#### Experimental series I: Engraftment of MDA_KDRab27a_ or MDA_KDTRAF3IP2_ results in reduced tumor growth

**Figure 4:**
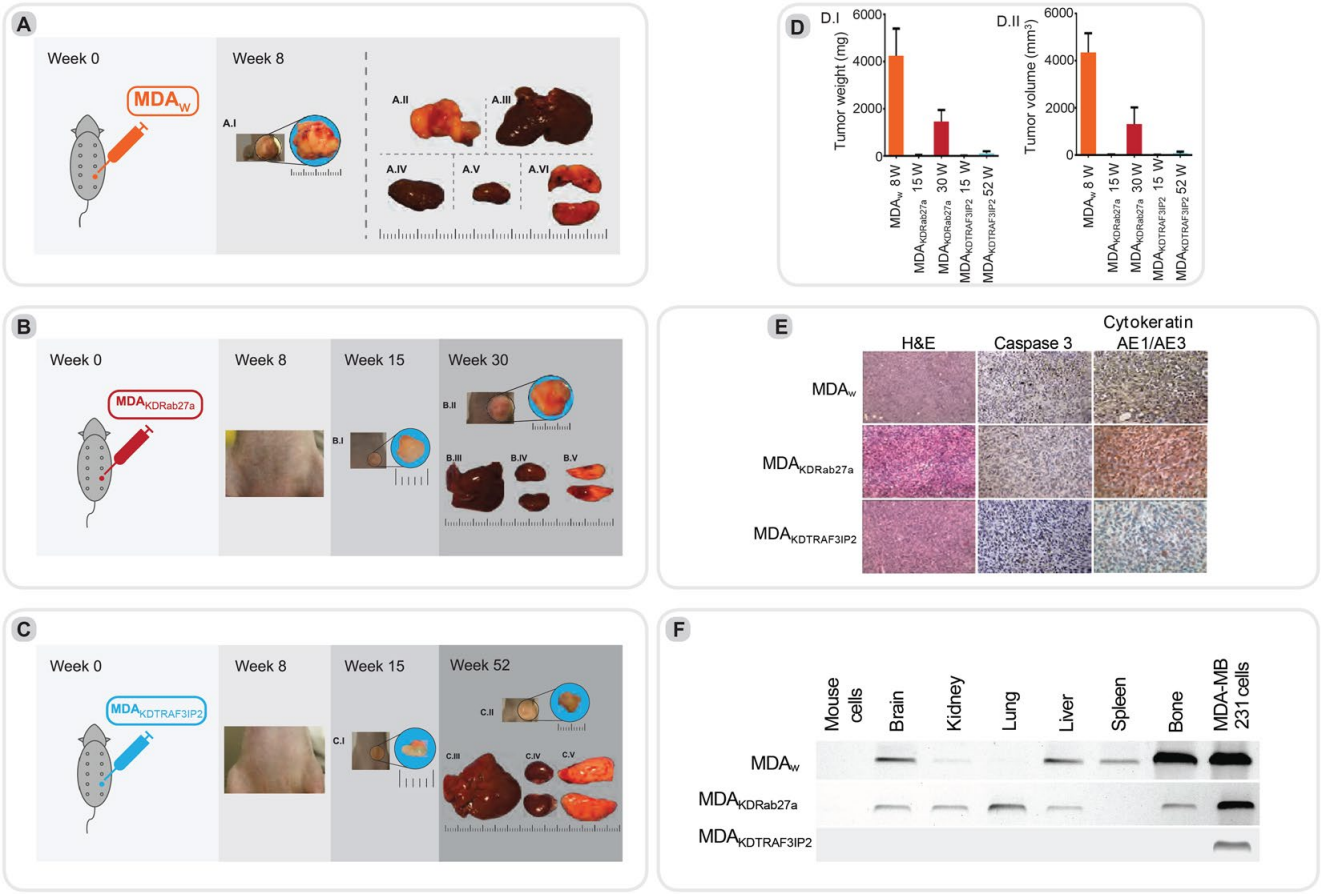
Experimental series I: (**A**) MDA-MB231 (MDA_w_) injected animals were euthanized eight weeks post-tumor induction. The abdominal metastasis as well as the internal organs are displayed in (A.II-A.VI B). MDA_KDRab27A_ injected animals were euthanized after 15 weeks (B.I) and 30 weeks (B.II) post-injection and were analyzed for tumor metastasis. No signs of macroscopic metastasis in major organs such as liver, kidneys, spleen and lungs (B.III-VI). **(C)** MDA_KDTRAF3IP2_ injected animals were euthanized 15 weeks (C.I) and 52 weeks (C.II) post-injection and were analyzed for metastasis. No macroscopic signs of metastasis were detected in any of the major organs (C.III-V). (**D**) Xenograft tumor weight and volume. Animals injected MDA_w_, MDA_KDTRAF3IP2_ and MDA_KDRab27a_ cells were sacrificed and tumors were isolated and weighted. Graph D.I illustrates tumor weight and D.II displays tumor volume in injected animals at different time points including the standard deviation. MDA_KDRab27a_-tumors are significantly less in size after 15 and 30 weeks of engraftment in comparison to the wild type. MDA_KDTRAF3IP2_ is significantly less in weight and volume after 15 as well as 52 weeks in comparison to MDA_w_ as well as MDA_KDRab27a_ after 30 weeks. (**E**) Histological analysis. After harvesting the tumors of MDA_w_, MDA_KDRab27a_ and MDA_KDTRAF3IP2_, tissue was stained for H&E, Caspase-3 and Cytokeratin AE1/AE2. H&E shows a very dense layer of cells in all three conditions. The breast cancer specific marker Cytokeratin AE1/AE2 is higher expressed in MDA_KDRab27a_ in in comparison to MDA_w_ and MDA_KDTRAF3IP2_. Caspase-3 is slightly increased in MDA_KDRab27a_ and MDA_KDTRAF3IP2_ tumors (**F**) Detection of micrometastasis. Tissues of the animals injected with MDA_w_, MDA_KDRab27a_ or MDA_KDTRAF3IP2_ were analyzed of micrometastasis. PCR analysis with primers directed towards a human-specific α-satellite DNA sequence of the centromere region of human chromosome 17 was used. Mouse cells extracted from healthy mice were used as a negative control. DNA of the human MDA-MB231 tumor cell line (MDA_w_) was used as positive control. Micrometastasis in most of the major organs of animals injected with MDA_w_ and MDA_KDRab27a_ was found. No signs of micrometastasis were found in mice injected with MDA_KDTRAF3IP2._ Displayed are cropped gels, for full gels see Supplementary Data.

Nude mice injected with MDA_w_ cells into 4^th^ left mammary gland developed large tumors in the primary location (average tumor weight: 4.2 g) at 8 weeks post-injection and displayed major metastases in abdomen, liver, bone, brain and spleen (Figs. 4AI–3AVI, Fig. 4F). However, no visible tumors were detected in animals injected with MDA_KDRab27a_ or MDA_KDTRAF3IP2_ cells at a similar time period (Fig. 4B,C). However, a very small tumor was detected after 15 weeks in 6 out of 6 animals injected with MDA_KDRab27a_ cells, but only 3 out of 6 mice injected with MDA_KDTRAF3IP2_ cells developed a small tumor. We then euthanized a subset of animals within each group at 15 weeks to analyze the tumors. The tumors exhibited limited growth in both MDA_KDRab27a_- and MDA_KDTRAF3IP2_-injected animals [average MDA_KDRab27a_ tumor weight at 15 weeks: ~0.017 g (P < 0.01 vs 4.2 g MDA_w_ tumor weight at 8 weeks, Supplementary Table 2), average MDA_KDTRAF3IP2_ tumor weight: ~0.004 g (P < 0.01 vs MDA_w_, Supplementary Table 2; Fig. 4 BI,CI)]. At 30 weeks post-injection, the experiment was terminated for the MDA_KDRab27a_-injected animals as the tumors grew [average tumor weight: ~1.4 g (P < 0.05 vs MDA_w_, Supplementary Table 2; Fig. 4BII-BV)]. At 52 weeks, the experiment was terminated to analyze tumor growth and metastasis in MDA_KDTRAF3IP2_ injected animals. Interestingly, this group showed no further tumor growth in comparison to MDA_w_- or MDA_KDRab27a_-injected animals [average tumor weight ~0.1 g (P < 0.01 vs MDA_w_, Supplementary Table 2; Fig. 4CII)]. A detailed statistical analysis is provided in Supplementary Table 2.

At necropsy, while MDA_w_ injected animals showed massive metastases in various organs (at week 8; Fig. 4AIII-AVI), there was no detectable metastasis in MDA_KDRab27a_ injected animals at 30 weeks (Fig. 4BIII-BV) or MDA_KDTRAF3IP2_ injected animals at 52 weeks post-injection (Fig. 4CIII-CV). Both tumor weight (Fig. 4DI) and tumor volume (Fig. 4DII) were significantly suppressed when Rab27a or TRAF3IP2 was silenced. However, qPCR for human specific sequence confirmed the presence of metastatic cells (micro-metastasis) in major organs of MDA_w_ (at week 8 post-injection) and MDA_KDRab27a_ injected animals (at week 30 post-injection), but not in MDA_KDTRAF3IP2_ injected animals after 52 weeks (Fig. 4F).

Histological analysis showed decreased expression of CytokeratinAE1/AE3 in MDA_KDTRAF3IP2_ compared to MDA_w_. However, the MDA_KDRab27a_ tumors showed higher expression of CytokeratinAE1/AE3 compared to MDA_w_ and MDA_KDTRAF3IP2_. The expression of Caspase-3 is slightly increased in MDA_KDRab27a_ and MDA_KDTRAF3IP2_ tumors (Fig. 4E).

#### Experimental series II: Silencing Rab27a or TRAF3IP2 affects communication between BC and stromal cells

**Figure 5:**
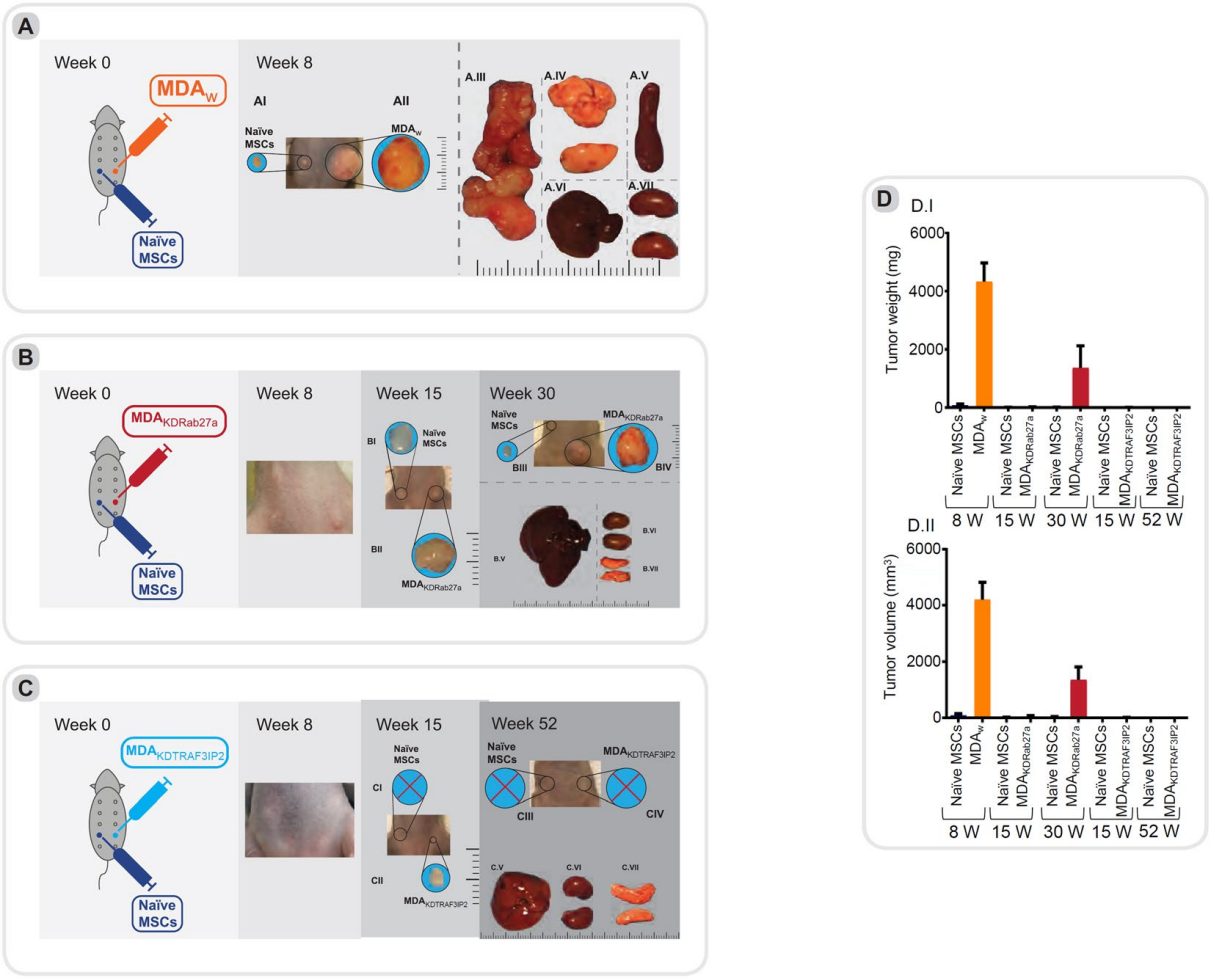
Experimental series II: Animals injected with MDA_w_ and naïve MSCs were euthanized eight weeks post-injection and analyzed for tumor metastasis of MSCs (A.I) and MDA_w_ cells (A.II). Metastasis was detected in the abdominal area (A.III). (**B**) naïve MSC + MDA_KDRab27a_: Animals injected with MDA_KDRab27a_ and naïve MSCs were euthanized 15 weeks (B.I/B.II) and 30 weeks (B.III/B.IV) post-injection. Tumor growth of MDA_KDRab27a_ (B.II/B.IV) expanded exponentially in comparison to MSCs (B.I/B.III). No macroscopic signs of macroscopic metastasis were detected in major organs such as liver, kidneys and lungs (B.V-VII). (**C**) naïve MSC + MDA_KDTRAF3IP2_: Animals injected with MDA_KDTRAF3IP2_ and naïve MSC were euthanized 15 weeks (C.I/C.II) and 52 weeks (C.III/C.IV) post-injection. After 15 weeks tumor growth was only seen on the side injected with MDA_KDTRAF3IP2_ (C.II), no tumor growth was shown on naïve MSC side (C.I). None of the mice showed tumor growth after 52 weeks (C.III/C.IV). No macroscopic signs of metastasis were detected in major organs (C.V-VII). (**D**) Xenograft tumor weight and volume. Animals injected MDA_w_/MSCs, MDA_KDRab27a_/MSCs or MDA_KDTRAF3IP2_/MSCs cells were sacrificed and tumors were isolated and weighted. Graph D.I illustrate tumor weight and D.II display tumor volume in injected animals at different time points including the standard deviation. All groups are significantly less in weight in comparison to MDA_w_. All groups, except MDA_w_, are significantly less in weight than MDA_KDRab27a_ after 30 weeks.

To evaluate the impact of silencing Rab27a and TRAF3IP2 in malignant breast cancer cells and their communication with MSCs, nude mice were injected with naïve MSCs into the 4^th^ right mammary gland and either MDA_w_, MDA_KDRab27a_ or MDA_KDTRAF3IP2_ cells in the contralateral mammary gland. The control group animals were injected with naïve MSCs into the 4^th^ mammary gland and MDA_w_ cells into the contralateral mammary gland. Animals in the control group showed a significant tumor growth on both sides after 8 weeks (~0.069 g on the naïve MSC-injected site: ~4.3 g at the MDA_w_-injected site; Supplementary Table 3; Fig. 5AI-II). Necropsy revealed massive metastasis in abdominal area of control group (Fig. 5AIII). In the group injected with naïve MSCs on one side and MDA_KDRab27a_ cells in the contralateral mammary gland, tumor growth was seen at both sites after 15 weeks [~0.004 g at the site injected with naïve MSCs (P < 0.001 vs. MDA_w_), average tumor weight of the injected with MDA_KDRab27a_ cells: ~0.012 g (P < 0.001 vs. MDA_w_), Supplementary Table 3; Fig. 5BI-II] and 30 weeks post tumor induction [average tumor weight of the injected with naïve MSCs: ~0.006 g, (P < 0.001 vs. MDA_w_), average tumor weight of the injected with MDA_KDRab27a_ cells: ~1.4 g, (P < 0.01 vs. MDA_w_), Fig. 5BIII-IV]. The weight of both tumors was comparable after 15 weeks, however, compared to the MDA_KDRab27a_ injected side, there was a significantly smaller tumor on the naïve MSC injected side after 30 weeks (Fig. 5BIII). No signs of macroscopic metastasis were found in major organs such as liver, kidneys or lungs (Fig. 5BV-VII).

In the third group of animals injected with naïve MSCs into 4th mammary gland and MDA_KDTRAF3IP2_ cells into the contralateral mammary gland, no tumor growth was detectable at the naïve MSC injected site after 15 (Fig. 5CI) and 52 weeks post-tumor induction (Fig. 5CIII). However, after 15 weeks a small tumor was detected in 2 out of 6 animals injected with MDA_KDTRAF3IP2_ [~0.002 g (P < 0.001 vs. MDA_w_), Supplementary Table 3; Fig. 5CII]. None of the mice showed detectable tumor growth (Fig. 4CIV) after 52 weeks. Also, at necropsy, no metastasis was detected in major organs (Fig. 5CV-VII).

In summary, tumors formed by MDA_w_ after 8 weeks were significantly heavier in comparison to MDA_KDRab27a_, MDA_KDTRAF3IP2_ or any tumor-like masses produced by MSCs at any time point. MDA_KDRab27a_ after 30 weeks was significantly heavier than any other tumor or tumor-like mass except MDA_w_. No tumor growth was seen in MDA_KDTRAF3IP2_ after 52 weeks as well as MSC coninjected with MDA_KDTRAF3IP2_ after 15 or 52 weeks. The detailed statistical analysis is displayed in Supplementary Table 3. The data indicate that regardless of the combination of injections without or with naïve MSCs, the tumorigenesis of MDA_KDRab27a_ and MDA_KDTRAF3IP2_ cells are significantly reduced (Fig. 5D).

#### Experimental series III: Regression of pre-existing breast tumors by lentiviral TRAF3IP2 shRNA

**Figure 6:**
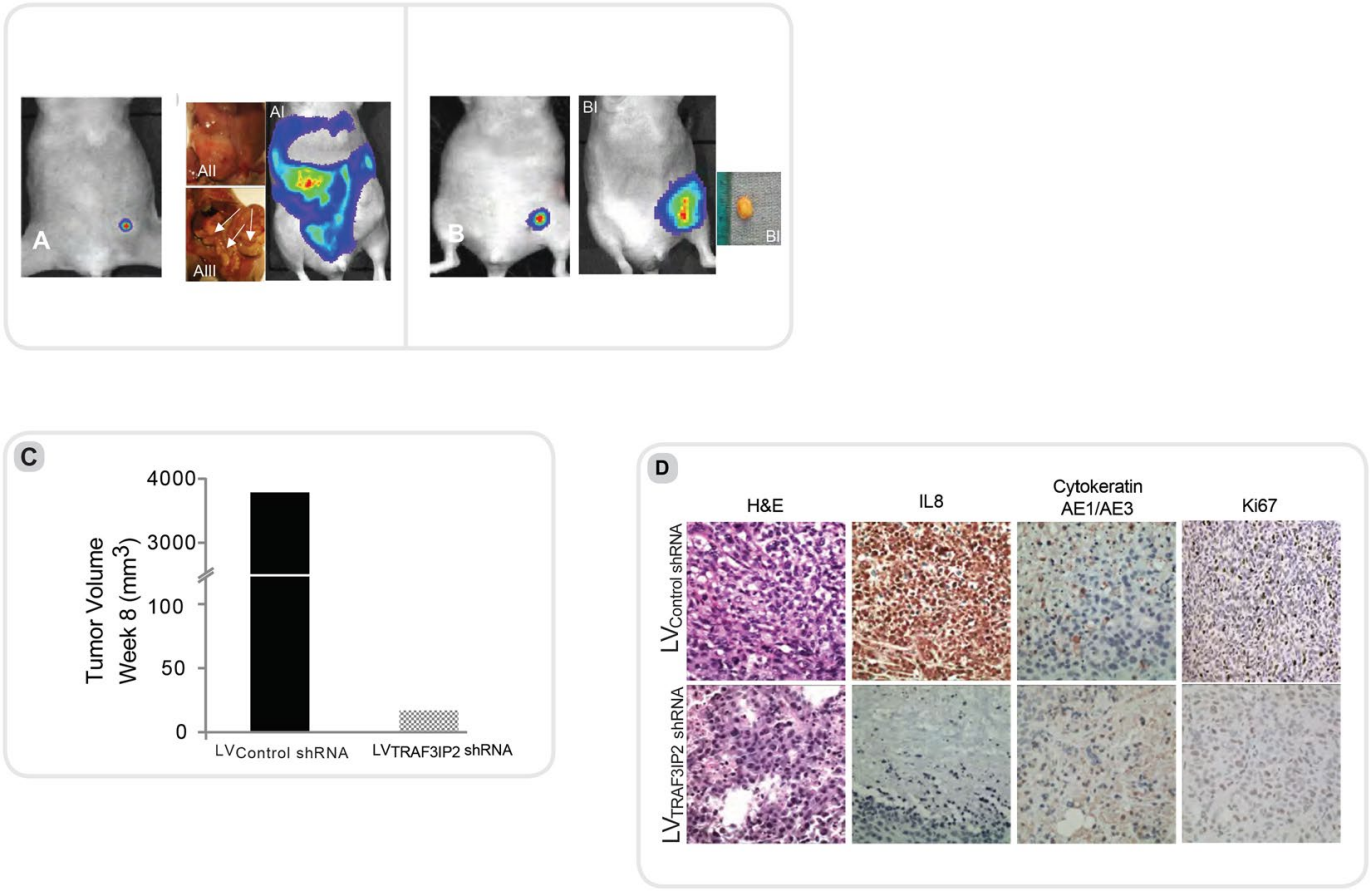
Experimental series III: Animals were injected with MDA_w_ and divided up after 14 days into two groups. The first group was weekly injected with LV_TRAF3IP2_ shRNA (**B**), the second group was injected an lentivirus containing scrambled shRNA (LV_Control_ shRNA) (**A**). Imaging for luciferase as well as post mortem analysis shows a significant reduction in tumor size of animals treated with TRAF3IP2 shRNA (**B**) in comparison to the control (**A**). (**C**) illustrates the tumor volume after 8 weeks. As seen in macroscopic pictures the volume of animals treated with TRAF3IP2 shRNA is significantly less than the control. (**D**) Histology analysis: After harvesting the tumors of mice treated with LV_TRAF3IP2_ KD or LV_Control shRNA_ tissue was stained for H&E, IL8 Cytokeratin AE1/AE2 and Ki67. H&E shows a dense layer of cells in both groups. IL8, Cytokeratin AE1/AE3 as well as Ki67 show a reduction after treatment with LV_KDTRAF3IP2_.

**Figure 7:**
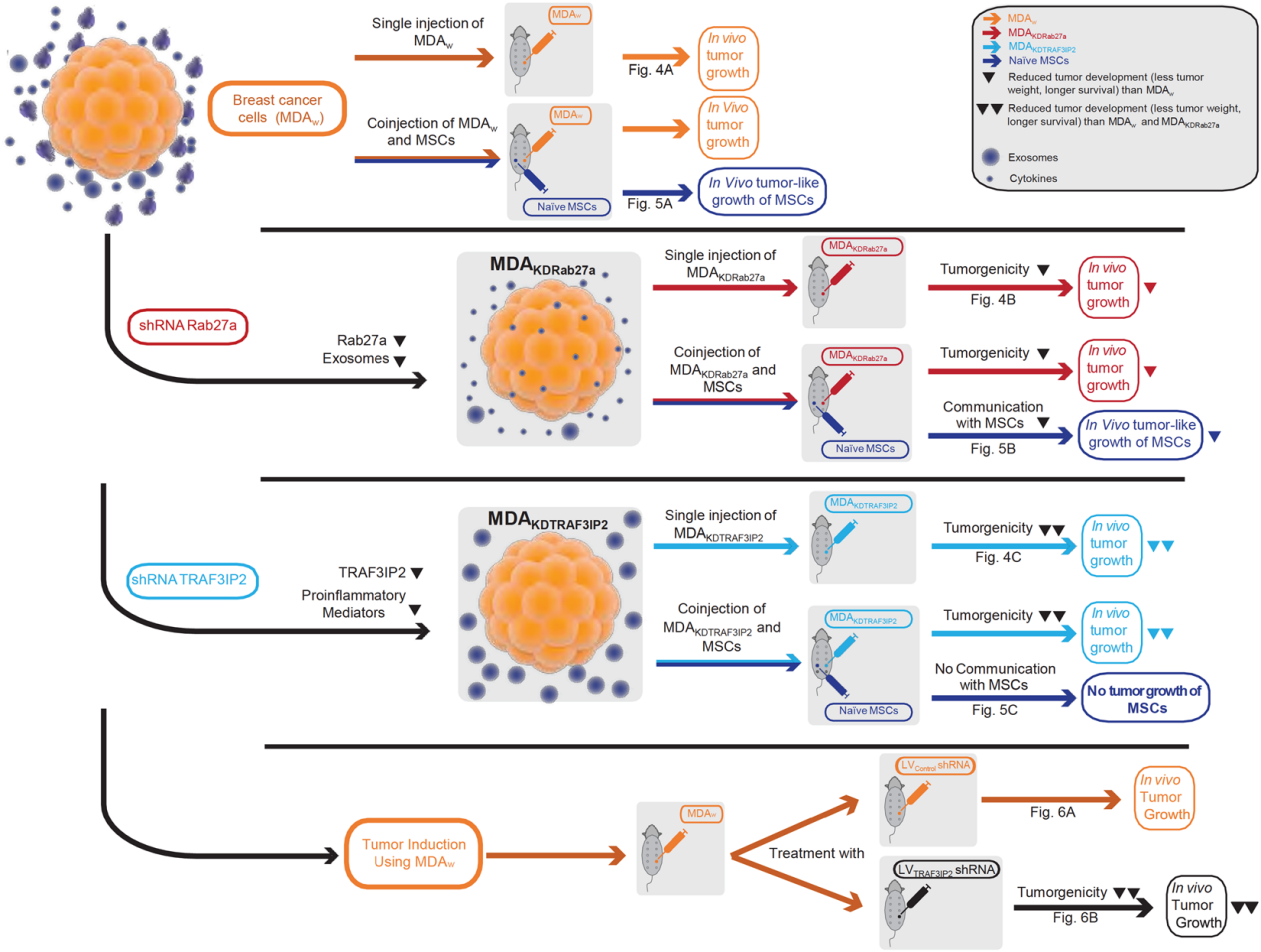
Schematic results of the experimental studies I, II and III are displayed. The top row shows the injection pattern of control group with MDA_w_, which resulted in significant tumor growth. After transduction with Rab27a shRNA we saw a reduction in Rab27a expression and exosome release (indicated by the reduction of large purple circles), resulting in reduced tumor growth of the tumor cells as well as MSC in comparison to MDA_w_. The third row shows the results of the injection MDA_KDTRAF3IP2_ in nude mice. Here we see reduced tumor growth in comparison to MDA_w_ and MDA_KDRab27a_ as well as the complete inhibition of communication with MSCs. The bottom row represents experimental series III, where primarily MDA_w_ cells were injected and then treated twice a week with either a lentiviral control (LV_Control_ shRNA) or a lentivirus TRAF3IP2 shRNA (LV_TRAF3IP2_ shRNA).

We next determined whether treating pre-existing tumors with lentiviral TRAF3IP2 shRNA regresses their size. The results in Fig. 6A,B show a marked reduction in tumor size after 8 weeks in TRAF3IP2_KD_ shRNA-LV treated mice versus scrambled shRNA-LV (Fig. 6A,B). While tumors grew continuously in scrambled shRNA-LV treated animals, the tumor growth was reduced in TRAF3IP2_KD_ shRNA-LV treated mice. At necropsy, the TRAF3IP2_KD_ shRNA-LV treated mice showed a small residual tumor (Fig. 6C), which upon analysis revealed a marked reduction in IL-8, CytokeratinAE1/AE3 and Ki67 expression (Fig. 6D). A detailed summary of the results is displayed in Fig. 7.

## Discussion

The results show that (1) silencing Rab27a or TRAF3IP2 in the triple negative breast cancer (BC) cell line MDA-MB231 results in the formation of a significantly smaller tumor in a breast xenograft model and significantly extends survival. Moreover, (2) while targeting Rab27a significantly *reduces* metastasis, targeting TRAF3IP2 *prevents* it. (3) Silencing Rab27a and TRAF3IP2 each inhibit interactions between breast cancer cells and naïve MSCs, resulting in reduced tumor growth in MSC-injected contralateral breasts. (4) More importantly, treatment of pre-existing tumors formed by the wildtype malignant BC cells with lentiviral TRAF3IP2 shRNA not only regressed tumor size, but also prevented metastasis. Together, these novel data suggest that TRAF3IP2 is a potent pro-tumorigenic mediator, and thus a potential target in breast cancer.

The role of Rab27a and TRAF3IP2 in many tumors, including breast cancer, is still unclear. Silencing of Rab27a has been shown to suppress gastric, pancreas and lung tumors^32–34^. In glioblastoma, we reported that silencing TRAF3IP2 is linked to reduced tumor growth and metastasis^35^. On the other hand, TRAF3IP2 overexpression in keratinocytes is shown to induce proliferation and tumor growth^36^. Interestingly in the present study, silencing Rab27a or TRAF3IP2 in the breast cancer cell line MDA-MB231 differentially regulated the expression of genes involved in tumor development, growth and metastasis (Fig. 2A). *CDH2* (*N-cadherin*) is a calcium-dependent adhesion molecule, and its increased expression suppresses pancreatic tumor growth^37^. The expression of *SERPINB5* is linked to high tumor grade, nodal metastasis and perineural invasion in invasive ductal carcinomas of the breast^38^. Our results show that targeting Rab27a and TRAF3IP2 each decreased *SERPINB5* expression, but enhanced that of *Cadherin 2*, in both MDA_KDRab27a_ and MDA_KDTRAF3IP2_ cells. The expression of *MCAM/CD146*, a suppressor of breast cancer cell invasion^39^, is higher in MDA_KDTRAF3IP2_ compared to MDA_KDRab27a_ and MDA_w_ cells. The expression of *CXCL12*, which is involved in breast cancer development and progression^40^, is also inhibited in both MDA_KDRab27a_ and MDA_KDTRAF3IP2_ cells. Furthermore, the proliferation of MDA_KDTRAF3IP2_ cells is significantly reduced, as evidenced by a significant  reduction in the expression of *TGFβ1* and *MAP2K5*, mediators involved in cell proliferation, metastasis, and death^41^.

Persistent expression and activation of MMPs play a role in inflammation and invasion. The expression of the endogenous MMP inhibitors *TIMP1* and *TIMP2*, which are involved in proliferation and inhibition of apoptosis^42^, is increased in MDA_KDRab27a_, but suppressed in MDA_KDTRAF3IP2_ cells. *MMP7* promotes tumor growth in many different cancers, including breast cancer^43^. Silencing TRAF3IP2 downregulated its expression in MDA-MB231 cells compared to MDA_w_ and MDA_KDRab27a_ cells. A correlative downregulation was found in the transcription factors, *ANGPTL4*, which is critical for invasion of MDA-MB231 cells^44^, and *ALDH3A1*, which promotes a multi-modality resistance in human breast adenocarcinomas^45^. *ANGPT1* (Angiopoetin 1), which is downregulated in MDA_KDTRAF3IP2_, is a key pro-angiogenic factor, like *VEGFA*, that enhances endothelial cell migration and the formation of capillary-like structures^46^. This broad alteration in gene expression suggests the upstream involvement of Rab27a and TRAF3IP2 within the cell. In summary, targeting TRAF3IP2 appears to have a bigger impact on downregulation of genes involved in inflammation and pro-tumorigenic pathways, compared to silencing Rab27a.

Interestingly, silencing Rab27a and TRAF3IP2 each induced morphological changes in MDA-MB231 cells. Electron microscopic analysis indicated that silencing Rab27a results in the formation of a porous cell surface in MDA_KDRab27a_ cells, while silencing TRAF3IP2 resulted in a rough surface, suggesting that Rab27a and TRAF3IP2 might be involved in remodeling or destabilization of tumor cell membrane. In addition, silencing Rab27a or TRAF3IP2 increased doubling time compared to the control MDA_w_ cells (Fig. 1B), possibly contributing to smaller tumors. However, silencing Rab27a or TRAF3IP2 had no significant effects on non-malignant cells like MSCs, suggesting that targeting Rab27aor TRAF3IP2 affect only the tumor cells but not the normal mesenchyml cells. Furthermore, the breast cancer cells can induce the expression of Rab27a or TRAF3IP2 in non-malignant breast epithelial 184A1 cells and naïve MSCs via a paracrine mechanism (Fig. 1A), and demonstrates the impact of breast cancer cells on surrounding non-malignant stroma.

TRAF3IP2 is an upstream regulator of NF-κB-dependent inflammatory signaling^47^. To understand the role of TRAF3IP2 on inflammatory responses, we silenced its expression in MDA-MB231 cells. The data show that TRAF3IP2 knockdown markedly suppressed IL11 expression. Since IL11 is involved in migration and invasion in MDA-MB231 cells (Fig. 1F)^31^, our results suggest that it could be one of the key factors involved in tumor growth seen in our *in vivo* data. Additionally, the expressions of several genes of the TGFβ superfamily, such as *BMP2*, *BMP3*, *BMP4*, *TGFβ2*, *TGFβ3*, *TNFRSF11B* as well as *INHα*, are differentially regulated by TRAF3IP2 silencing. The TGFβ superfamily has been shown exert dual effects; it is shown to either suppress or promote tumor growth^48^. Our data show downregulation of *BMP2* and *BMP3*. Expression of *BMP2* is closely related to invasion of breast cancer cells by cytoskeletal reorganization and decreased adhesion^49^. Expression of *BMP3* has been shown to positively correlate with MSC proliferation^50^. The function of *BMP4*, whose expression is higher in MDA_KDTRAF3IP2_, is not fully understood. While a number of studies have shown its pro-growth effects in other cancers^51^, its increased expression has shown to inhibit breast cancer growth^52^. The expression of TGFβ3, which is lower in TRAF3IP2-silenced MBA-MB231 cells has been linked to enhanced metastasis in breast carcinoma^46^. The expression of *TNFRSF11B* and *INHα* are enhanced in both MDA_KDRab27a_ and MDA_KDTRAF3IP2_ cells. Downregulation of *TNFRSF11B* (TNFR1) correlates with tumorgenicity and poorer prognosis in patients with breast cancer^53^. *INHα* has been suggested to have a tumor suppressor effect by suppression of cell growth and being associated with apoptosis^1^. *PDGFA* is related to tumor progression in breast cancer^54^, and its expression is reduced in MDA_KDTRAF3IP2_. However, a small increase in the expression of *FIGF* (VEGFD) and CSF1 (Colony stimulating factor 1) was observed in MDA_KDTRAF3IP2_ cells. FIGF, a VEGF family member, is, if expressed, involved in lymphangiogenesis in breast cancer^55^ but also has been shown to be downregulated in a metastic breast cancer cells line^56^. CSF1 is involved in breast cancer progression^57^. The *LTα* gene, associated with tumor progression and angiogenesis in cutaneous lymphomas, shows a significantly reduction in its expression in MDA_KDTRAF3IP2_^58^. In accordance to our previous experiment here we display the profound impact of TRAF3IP2 within the cells. Many genes involved in cancer development and progression are altered in a tumor suppressive matter in MDA_KDTRAF3IP2_.

Supporting our *in vitro* data, the *in vivo* data demonstrate that silencing Rab27a delays tumor development but not micrometastasis. However, targeting Rab27a significantly extended survival by more than 3-fold compared to the wild type. on the other hand, targeting TRAF3IP2 suppressed both tumor growth as well as macro- and micrometastasis. Furthermore, targeting Rab27a or TRAF3IP2 each reduced the potential of the tumor cells with the surrounding stroma, indicated by reduced growth of MSCs coinjected with MDA_KDRab27a_. Coinjection of MDA_KDTRAF3IP2_ and naïve MSCs resulted no tumor growth on MSC injected side, indicating lack of interaction between the MDA_KDTRAF3IP2_ and naïve MSCs. More importantly, targeting TRAF3IP2 not only regressed pre-formed tumors (Fig. 6), but also prevented metastasis. One potential explanation of reduced effect of Rab27a could be that silencing Rab27 blunts, but does not abrogate exosome release^8,59^. In fact, histologic analysis revealed a higher expression of Cytokeratin AE1/AE3 in MDA_KDRab27a_ in comparison with MDA_w_ or MDA_KDTRAF3IP2_. This is in accordance with previous findings where exosomes of MDA-MB231 cells have been shown to contain cytokeratin 9 and that the reduction of Rab27a results in the intracellular accumulation of enlarged MVEs^59,60^. As indicated by our data, several genes altered by silencing TRAF3IP2 are individually the subject of clinical trials. For example, BMP4 is currently being tested as potential treatment for glioblastoma^61^, FIGF in end stage coronary heart disease^62^, and a MAP kinase inhibitor in non-small cell lung cancer^63^.

A limitation of the present study is that we used a single breast cancer cell line to investigate potential roles of silencing Rab27a and TRAF3IP2 on tumor growth. Our future studies will involve the use of patient-derived xenotransplants to further validate these fundamental first *in vivo* and *in vitro* results.

## Conclusions

Both Rab27a and TRAF3IP2 play a causal role in breast cancer growth and metastasis. We showed that targeting Rab27a decreases exosome release and delays progression of tumor growth, but fails to affect micrometastasis. Targeting TRAF3IP2, as a representative of the soluble fraction of the TME, however, suppresses tumor growth as well as macro- and micro-metastasis. More importantly, treatment with lentiviral TRAF3IP2 shRNA regresses pre-formed tumors and prevents metastasis. These results indicate that TRAF3IP2 is a more potent inhibitor of tumorigenesis, and thus a novel therapeutic target in breast cancer.

## Supplementary information

Supplementary Figures.

Supplementary Tables.

## References

[1] Siegel RL, Miller KD, Jemal A (2019). Cancer statistics, 2019. CA Cancer J. Clin..

[2] Hu M, Polyak K (2008). Microenvironmental regulation of cancer development. Curr. Opin. Genet. Dev..

[3] Li W (2015). Gastric cancer-derived mesenchymal stem cells prompt gastric cancer progression through secretion of interleukin-8. J. Exp. Clin. Cancer Res..

[4] Mao Y, Keller ET, Garfield DH, Shen K, Wang J (2013). Stromal cells in tumor microenvironment and breast cancer. Cancer Metastasis Rev..

[5] Senst C (2013). Prospective dual role of mesenchymal stem cells in breast tumor microenvironment. Breast Cancer Res. Treat..

[6] Worner PM (2019). Breast Tumor Microenvironment Can Transform Naive Mesenchymal Stem Cells into Tumor-Forming Cells in Nude Mice. Stem Cell Dev..

[7] Kruger S (2014). Molecular characterization of exosome-like vesicles from breast cancer cells. BMC Cancer.

[8] Bobrie A, Colombo M, Raposo G, Thery C (2011). Exosome secretion: molecular mechanisms and roles in immune responses. Traffic.

[9] Wang S (2019). Exosomes secreted by mesenchymal stromal/stem cell-derived adipocytes promote breast cancer cell growth via activation of Hippo signaling pathway. Stem Cell Res. Ther..

[10] Biswas Subir, Mandal Gunjan, Roy Chowdhury Sougata, Purohit Suman, Payne Kyle K., Anadon Carmen, Gupta Arnab, Swanson Patricia, Yu Xiaoqing, Conejo-Garcia José R., Bhattacharyya Arindam (2019). Exosomes Produced by Mesenchymal Stem Cells Drive Differentiation of Myeloid Cells into Immunosuppressive M2-Polarized Macrophages in Breast Cancer. The Journal of Immunology.

[11] Lin R, Wang S, Zhao RC (2013). Exosomes from human adipose-derived mesenchymal stem cells promote migration through Wnt signaling pathway in a breast cancer cell model. Mol. Cell Biochem..

[12] Miller IV, Grunewald TG (2015). Tumour-derived exosomes: Tiny envelopes for big stories. Biol. Cell.

[13] Bobrie A (2012). Rab27a supports exosome-dependent and -independent mechanisms that modify the tumor microenvironment and can promote tumor progression. Cancer Res..

[14] Mantovani A (2010). The chemokine system in cancer biology and therapy. Cytokine Growth Factor. Rev..

[15] Kesanakurti D, Chetty C, Rajasekhar Maddirela D, Gujrati M, Rao JS (2013). Essential role of cooperative NF-kappaB and Stat3 recruitment to ICAM-1 intronic consensus elements in the regulation of radiation-induced invasion and migration in glioma. Oncogene.

[16] Katanov C (2015). Regulation of the inflammatory profile of stromal cells in human breast cancer: prominent roles for TNF-alpha and the NF-kappaB pathway. Stem Cell Res. Ther..

[17] Anthony NG (2017). Inhibitory Kappa B Kinase alpha (IKKalpha) Inhibitors That Recapitulate Their Selectivity in Cells against Isoform-Related Biomarkers. J. Med. Chem..

[18] Hunter CA (2007). Act1-ivating IL-17 inflammation. Nat. Immunol..

[19] Izadpanah R (2006). Biologic properties of mesenchymal stem cells derived from bone marrow and adipose tissue. J. Cell Biochem..

[20] Dominici M (2006). Minimal criteria for defining multipotent mesenchymal stromal cells. The International Society for Cellular Therapy position statement. Cytotherapy.

[21] Izadpanah R (2008). Long-term *in vitro* expansion alters the biology of adult mesenchymal stem cells. Cancer Res..

[22] Izadpanah R (2005). Characterization of multipotent mesenchymal stem cells from the bone marrow of rhesus macaques. Stem Cell Dev..

[23] Freisinger E (2010). Characterization of hematopoietic potential of mesenchymal stem cells. J. Cell Physiol..

[24] Molloy AP (2009). Mesenchymal stem cell secretion of chemokines during differentiation into osteoblasts, and their potential role in mediating interactions with breast cancer cells. Int. J. Cancer.

[25] Keller S, Sanderson MP, Stoeck A, Altevogt P (2006). Exosomes: from biogenesis and secretion to biological function. Immunol. Lett..

[26] VanGuilder HD, Vrana KE, Freeman WM (2008). Twenty-five years of quantitative PCR for gene expression analysis. Biotechniques.

[27] Stolzing A, Jones E, McGonagle D, Scutt A (2008). Age-related changes in human bone marrow-derived mesenchymal stem cells: consequences for cell therapies. Mech. Ageing Dev..

[28] Simpson RJ, Jensen SS, Lim JW (2008). Proteomic profiling of exosomes: current perspectives. Proteomics.

[29] Becker M (2002). Sensitive PCR method for the detection and real-time quantification of human cells in xenotransplantation systems. Br. J. Cancer.

[30] Yu S, Cao H, Shen B, Feng J (2015). Tumor-derived exosomes in cancer progression and treatment failure. Oncotarget.

[31] Lim JH (2014). Inhibition of the Interleukin-11-STAT3 Axis Attenuates Hypoxia-Induced Migration and Invasion in MDA-MB-231 Breast Cancer Cells. Korean J. Physiol. Pharmacol..

[32] Li J, Jin Q, Huang F, Tang Z, Huang J (2017). Effects of Rab27A and Rab27B on Invasion, Proliferation, Apoptosis, and Chemoresistance in Human Pancreatic Cancer Cells. Pancreas.

[33] Li X (2017). Effects of silencing Rab27a gene on biological characteristics and chemosensitivity of non-small cell lung cancer. Oncotarget.

[34] Li, Y. *et al*. miR-182-5p improves the viability, mitosis, migration, and invasion ability of human gastric cancer cells by down-regulating RAB27A. *Biosci Rep* **37**, 10.1042/BSR20170136 (2017). 10.1042/BSR20170136 PMC6434084 28546229

[35] Alt EU (2018). TRAF3IP2, a novel therapeutic target in glioblastoma multiforme. Oncotarget.

[36] Wu L (2015). A novel IL-17 signaling pathway controlling keratinocyte proliferation and tumorigenesis via the TRAF4-ERK5 axis. J. Exp. Med..

[37] Su Y, Li J, Shi C, Hruban RH, Radice GL (2016). N-cadherin functions as a growth suppressor in a model of K-ras-induced PanIN. Oncogene.

[38] Helal DS, El-Guindy DM (2017). Maspin expression and subcellular localization in invasive ductal carcinoma of the breast: Prognostic significance and relation to microvessel density. J. Egypt. Natl Canc Inst..

[39] Ouhtit A, Abdraboh ME, Hollenbach AD, Zayed H, Raj MHG (2017). CD146, a novel target of CD44-signaling, suppresses breast tumor cell invasion. Cell Commun. Signal..

[40] Chung B (2017). Human brain metastatic stroma attracts breast cancer cells via chemokines CXCL16 and CXCL12. NPJ Breast Cancer.

[41] Mishra AK, Parish CR, Wong ML, Licinio J, Blackburn AC (2017). Leptin signals via TGFB1 to promote metastatic potential and stemness in breast cancer. Plos One.

[42] Fernandez-Garcia B (2014). Expression and prognostic significance of fibronectin and matrix metalloproteases in breast cancer metastasis. Histopathology.

[43] Takahara T (2017). SIPA1 promotes invasion and migration in human oral squamous cell carcinoma by ITGB1 and MMP7. Exp. Cell Res..

[44] Adhikary T (2013). Inverse PPARbeta/delta agonists suppress oncogenic signaling to the ANGPTL4 gene and inhibit cancer cell invasion. Oncogene.

[45] Voulgaridou GP (2016). Aldehyde dehydrogenase 3A1 promotes multi-modality resistance and alters gene expression profile in human breast adenocarcinoma MCF-7 cells. Int. J. Biochem. Cell Biol..

[46] Flores-Perez A (2016). Dual targeting of ANGPT1 and TGFBR2 genes by miR-204 controls angiogenesis in breast cancer. Sci. Rep..

[47] Li X (2000). Act1, an NF-kappa B-activating protein. Proc. Natl Acad. Sci. USA.

[48] Ikushima H, Miyazono K (2010). TGFbeta signalling: a complex web in cancer progression. Nat. Rev. Cancer.

[49] Jin H (2012). BMP2 promotes migration and invasion of breast cancer cells via cytoskeletal reorganization and adhesion decrease: an AFM investigation. Appl. Microbiol. Biotechnol..

[50] Cernea M, Tang W, Guan H, Yang K (2016). Wisp1 mediates Bmp3-stimulated mesenchymal stem cell proliferation. J. Mol. Endocrinol..

[51] Ampuja M (2016). The impact of bone morphogenetic protein 4 (BMP4) on breast cancer metastasis in a mouse xenograft model. Cancer Lett..

[52] Cao Y (2014). BMP4 inhibits breast cancer metastasis by blocking myeloid-derived suppressor cell activity. Cancer Res..

[53] Shao Q (2017). Phospholipase Cdelta1 suppresses cell migration and invasion of breast cancer cells by modulating KIF3A-mediated ERK1/2/beta- catenin/MMP7 signalling. Oncotarget.

[54] Carvalho I, Milanezi F, Martins A, Reis RM, Schmitt F (2005). Overexpression of platelet-derived growth factor receptor alpha in breast cancer is associated with tumour progression. Breast Cancer Res..

[55] Zhu C, Qi X, Zhou X, Nie X, Gu Y (2016). Sulfatase 2 facilitates lymphangiogenesis in breast cancer by regulating VEGF-D. Oncol. Rep..

[56] Roberti MP (2012). Protein expression changes during human triple negative breast cancer cell line progression to lymph node metastasis in a xenografted model in nude mice. Cancer Biol. Ther..

[57] Ding J (2016). CSF1 is involved in breast cancer progression through inducing monocyte differentiation and homing. Int. J. Oncol..

[58] Lauenborg B (2015). Malignant T cells express lymphotoxin alpha and drive endothelial activation in cutaneous T cell lymphoma. Oncotarget.

[59] Ostrowski Matias, Carmo Nuno B., Krumeich Sophie, Fanget Isabelle, Raposo Graça, Savina Ariel, Moita Catarina F., Schauer Kristine, Hume Alistair N., Freitas Rui P., Goud Bruno, Benaroch Philippe, Hacohen Nir, Fukuda Mitsunori, Desnos Claire, Seabra Miguel C., Darchen François, Amigorena Sebastian, Moita Luis F., Thery Clotilde (2009). Rab27a and Rab27b control different steps of the exosome secretion pathway. Nature Cell Biology.

[60] Green TM, Alpaugh ML, Barsky SH, Rappa G, Lorico A (2015). Breast Cancer-Derived Extracellular Vesicles: Characterization and Contribution to the Metastatic Phenotype. Biomed. Res. Int..

[61] DiMeco, F. A Dose Escalation Phase I Study Of Human- Recombinant Bone Morphogenetic Protein 4 Administrated Via CED In GBM Patients, https://clinicaltrials.gov/ct2/show/record/NCT02869243#wrapper (2019).

[62] Hartikainen, J. EndocardialVascularEndothelialGrowth Factor D(VEGF-D)Gene Therapy for the Treatment of Severe Coronary Heart Disease (KAT301), https://clinicaltrials.gov/ct2/show/record/NCT01002430?term=figf&rank=1 (2009).

[63] Rajan, A. Randomized Phase II Study of AZD6244 MEK-Inhibitor With Erlotinib in KRAS Wild Type and KRAS Mutant Advanced Non-Small Cell Lung CancerArun Rajan, https://clinicaltrials.gov/ct2/show/record/NCT01229150 (2010).

