# Supplementary material for: Targeting TRAF3IP2, Compared to Rab27, is More Effective in Suppressing the Development and Metastasis of Breast Cancer
Source: Sci Rep. 2020 Jun 1;10:8834. doi: 10.1038/s41598-020-64781-z (PMC7264196; doi:10.1038/s41598-020-64781-z)
Supplement: Supplementary file 1 — Supplementary Figures. [file 41598_2020_64781_MOESM1_ESM.pdf]

Supplementary Figures

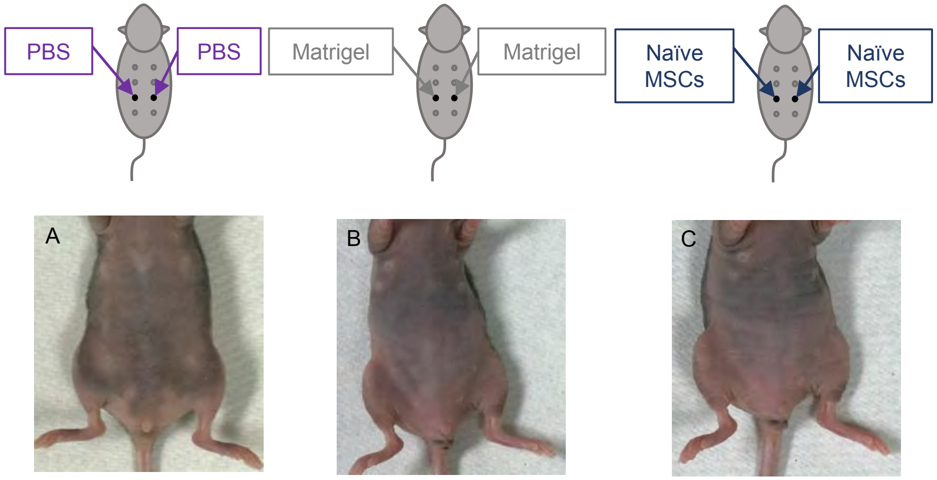

Supplementary Figure S1. Control groups for the in vivo experiments. Animals coinjected on contralateral sides with PBS (A), Matrigel (B), naïve MSCs (C) served as controls and monitored for tumor growth for up to 52 weeks. No tumor growth was seen.

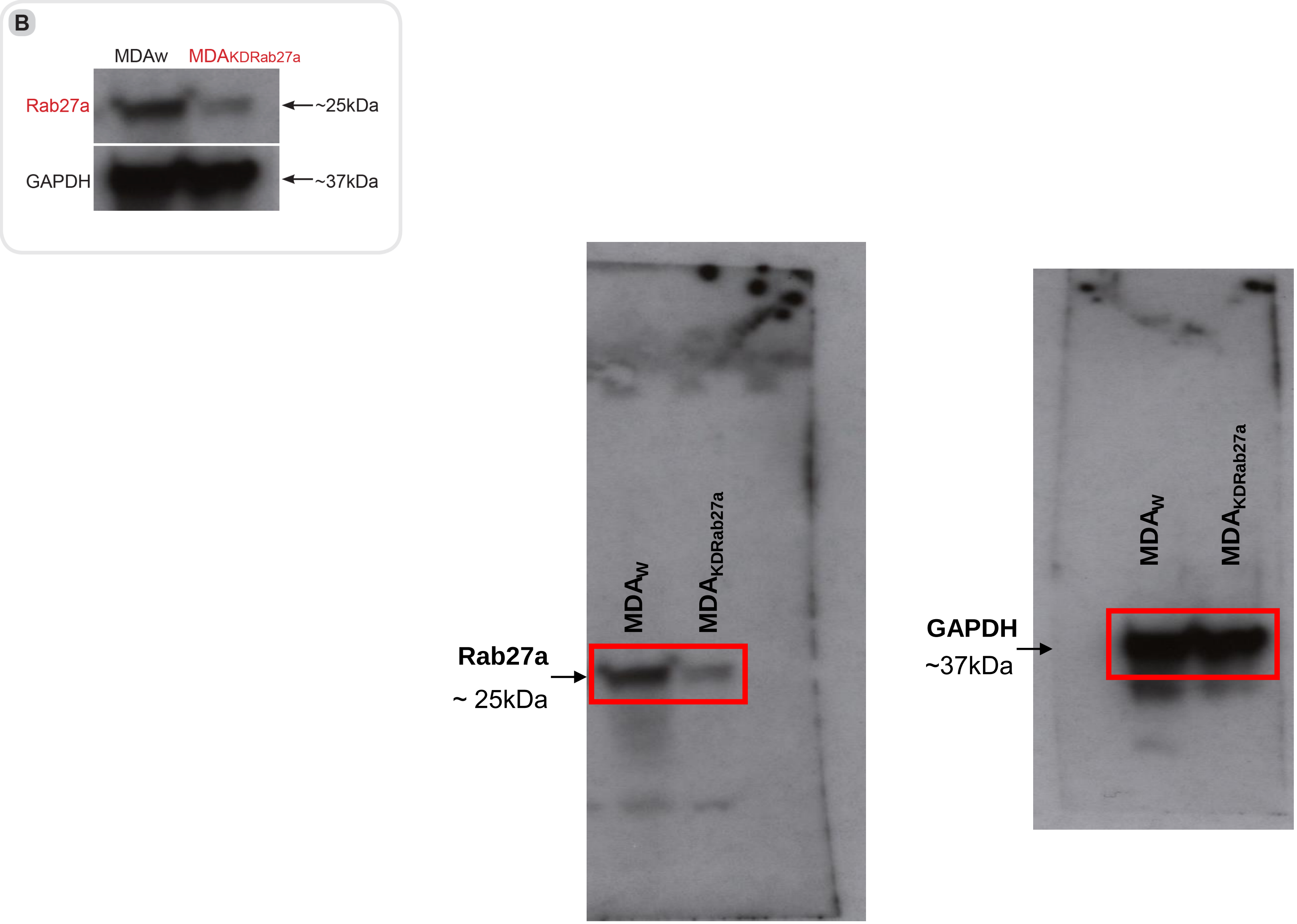

Supplementary Figure S2. Displayed is the full-length western blots of Figure 1B. The cropped picture of figure 1B is displayed on the top left corner. The experiment was performed on MDA-MB231 (MDA_w) and $MDA_{KDRab27a}$ cells stained with antibodies for Rab27a or GAPDH.

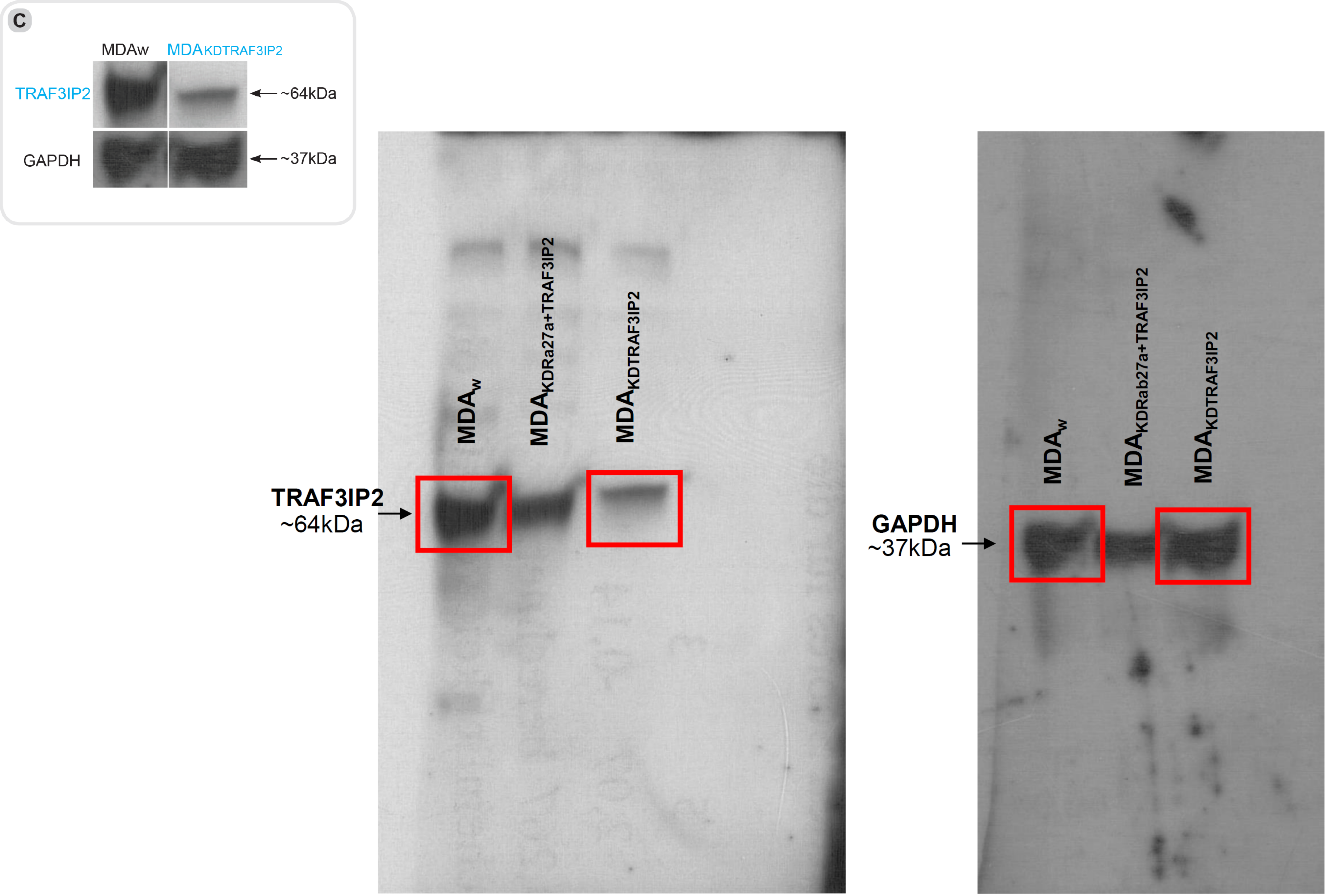

Supplementary Figure S3. Displayed is the full-length western blots of Figure 1C. The cropped picture of figure 1C is displayed on the top left corner. The experiment was performed on of MDA-MB231 (MDA_{w}), $MDA_{KDRab27a+TRAF3IP2}$ and $MDA_{KDTRAF3IP2}$ cells stained with antibodies for TRAF3IP2 or GAPDH. At the time of writing this manuscript the results of $MDA_{KDRab27a+TRAF3IP2}$ were due to an insufficient knockdown not used.

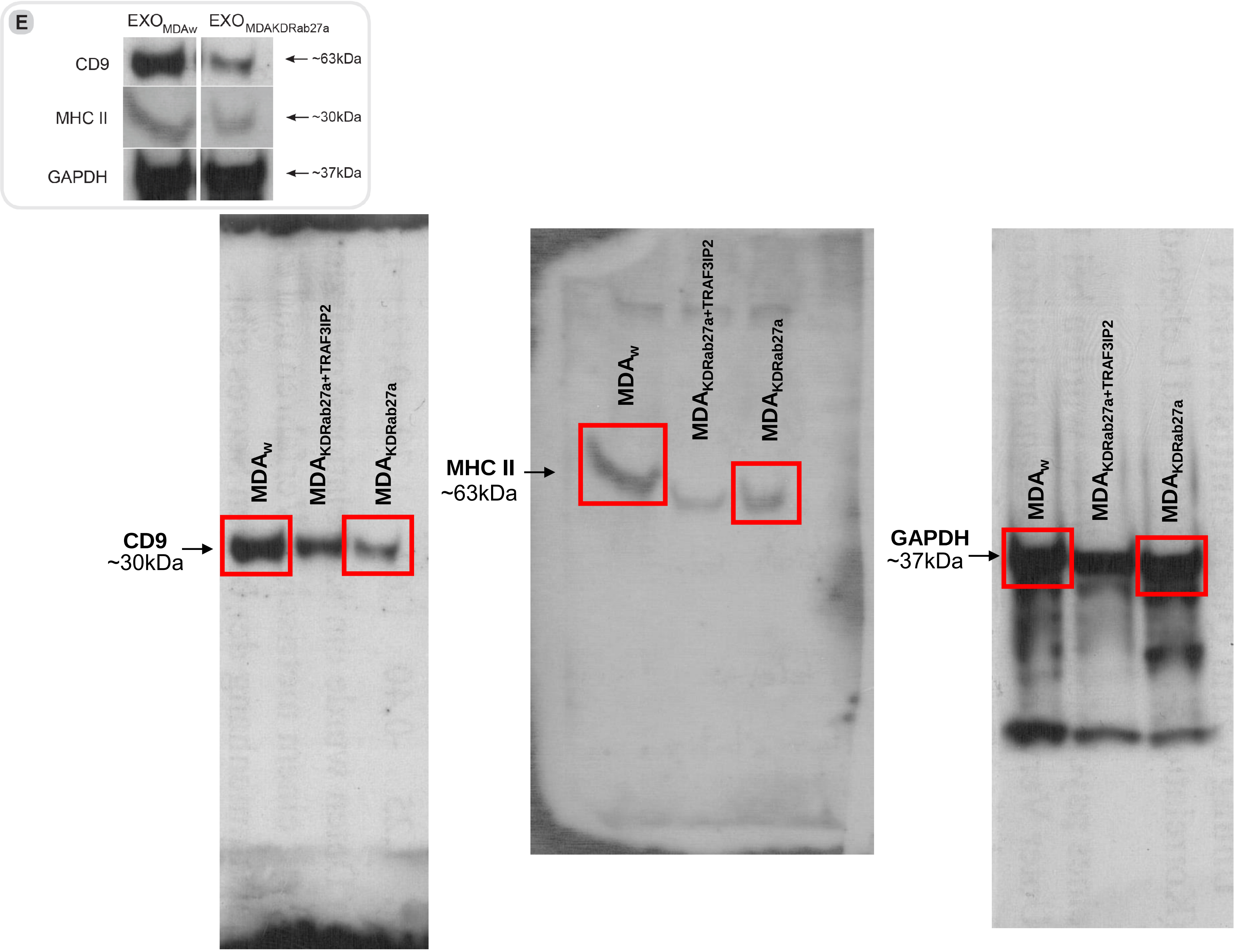

Supplementary Figure S4. Displayed is the full-length western blots of Figure 1.E. The cropped picture of figure 1E is displayed on the top left corner. The experiment was performed using condition media of MDA_{w}, $MDA_{KDRab27a+TRAF3IP2}$ and $MDA_{KDRab27a}$ using CD9 and MHCII as marker for exosomes as well as GAPDH as housekeeping gene. At the time of writing this manuscript the results of $MDA_{KDRab27a+TRAF3IP2}$ were due to an insufficient knockdown not used.

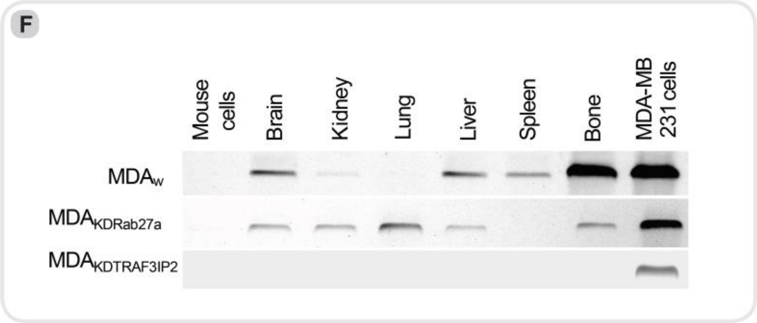

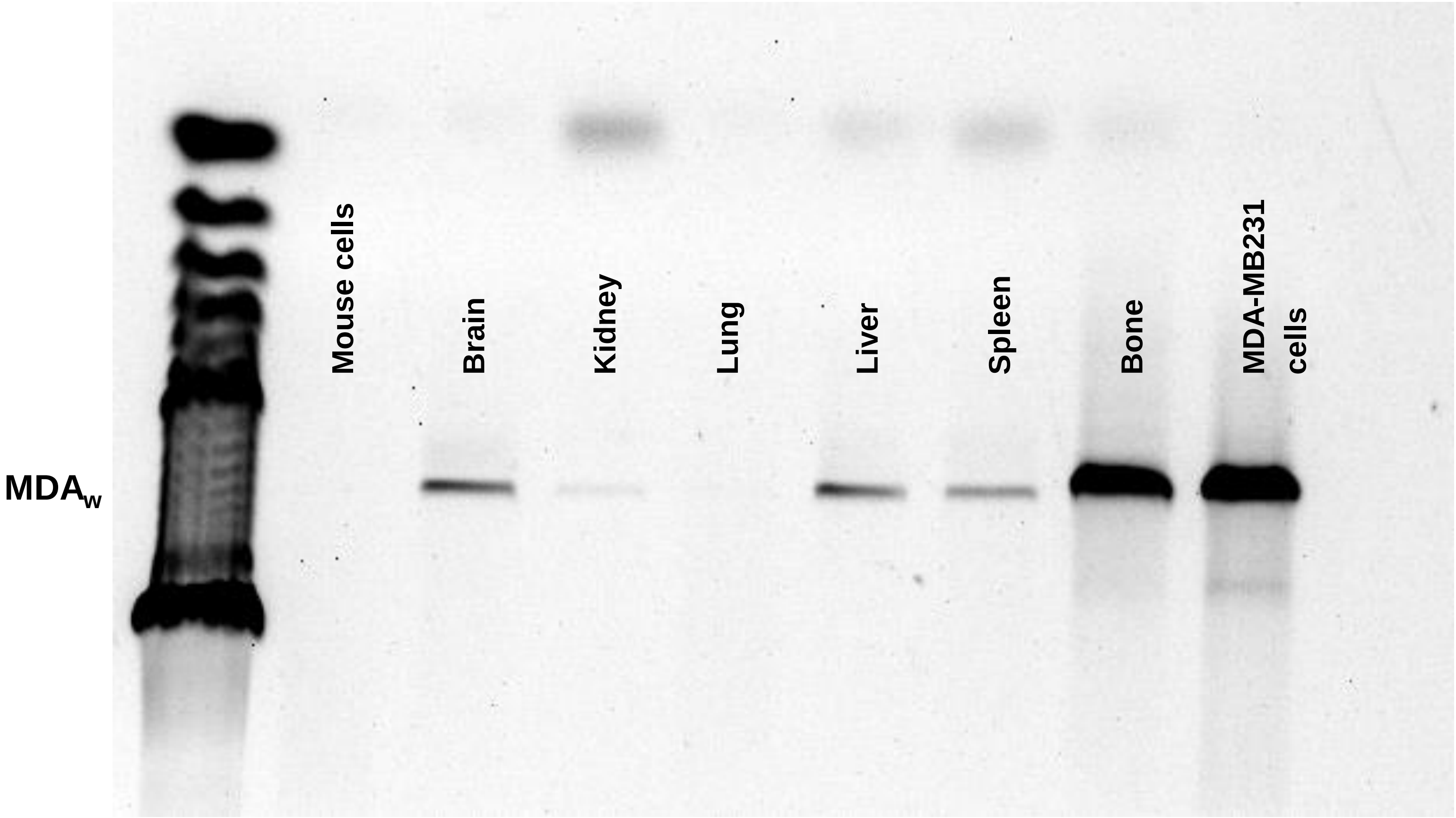

Supplementary Figure S5. Displayed is the full-length gel electrophoresis of DNA from MDA_w induced tumors of Figure 4F. Mouse cells extracted from healthy mice were used as a negative control. DNA of the human MDA-MB231 tumor cell line (MDA_w) was used as positive control.

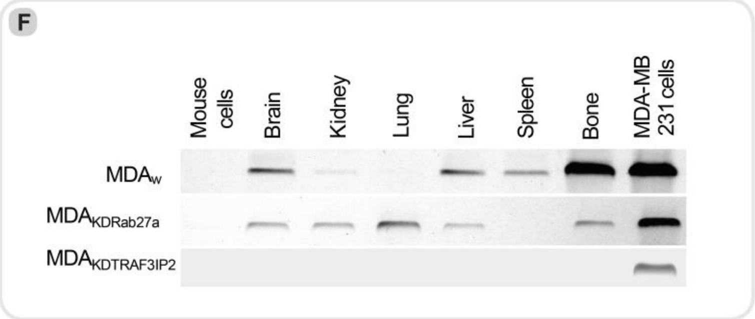

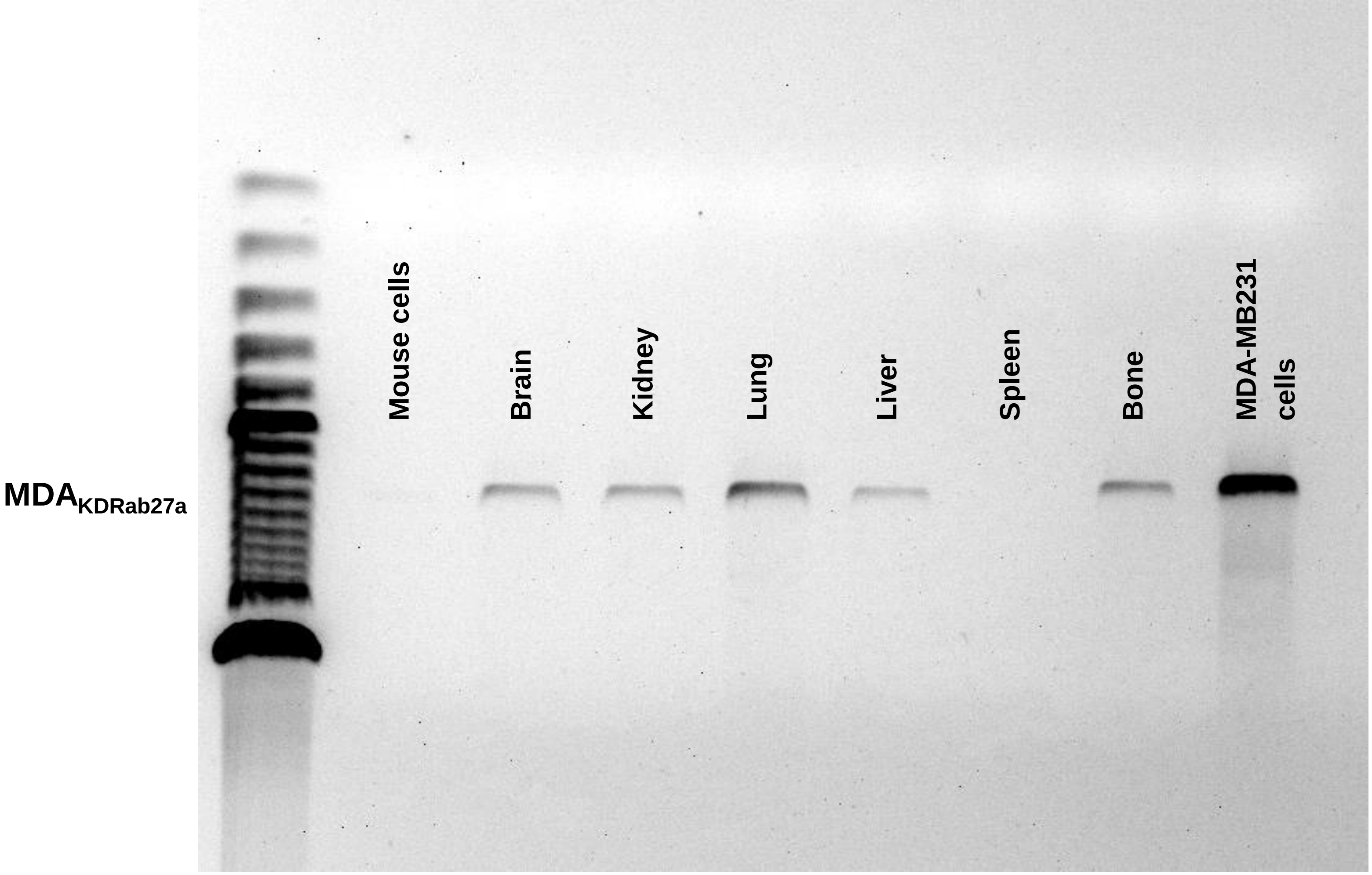

Supplementary Figure S6. Displayed is the full-length gel electrophoresis of DNA from $MDA_{KDRab27a}$ induced tumors of Figure 4F. Mouse cells extracted from healthy mice were used as a negative control. DNA of the human MDA-MB231 tumor cell line (MDA_w) was used as positive control.

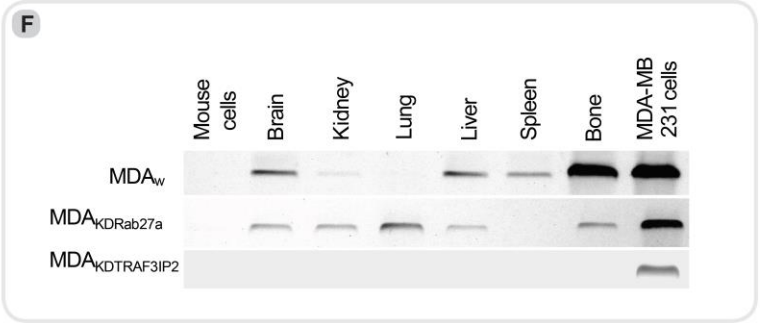

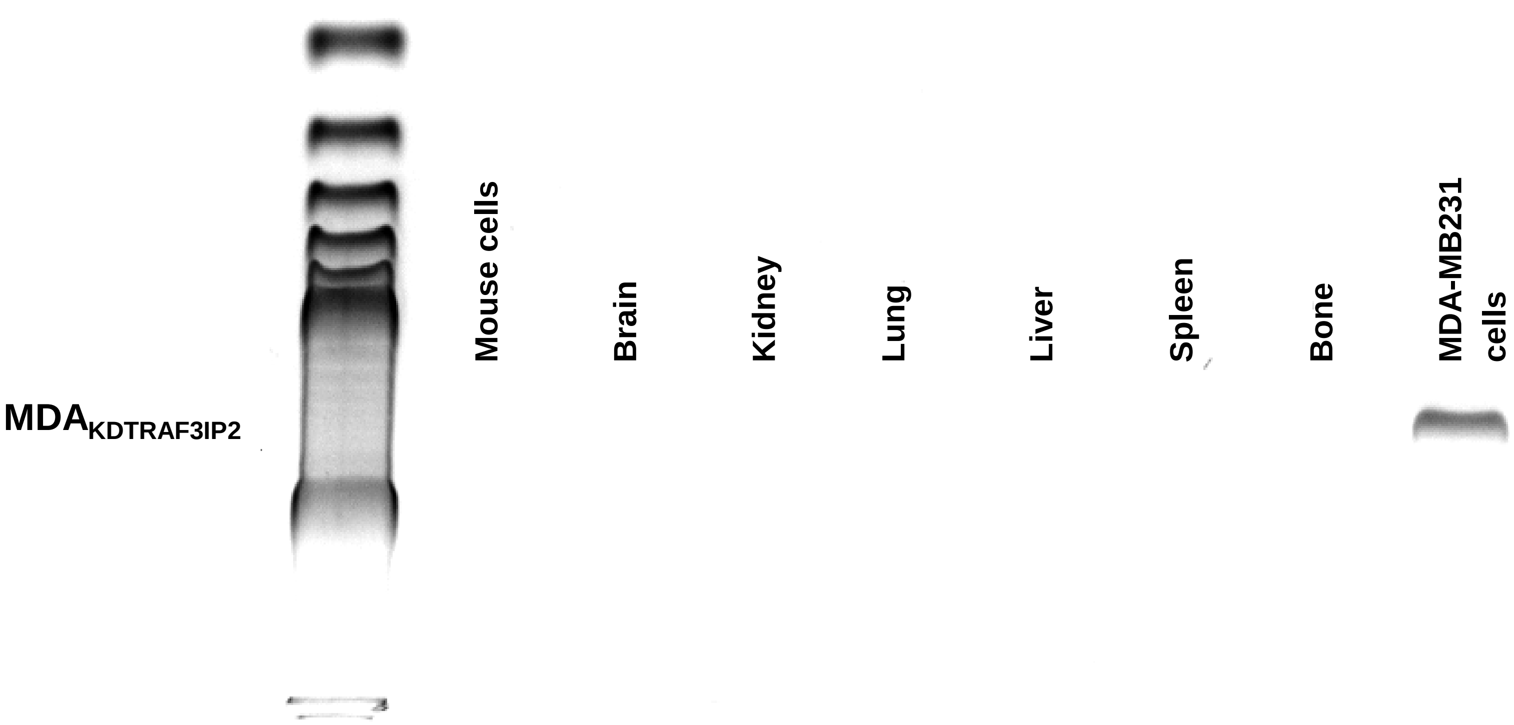

Supplementary Figure S7. Displayed is the full-length gel electrophoresis of DNA from $MDA_{KDTRAF3IP2}$ induced tumors of Figure 4F. Mouse cells extracted from healthy mice were used as a negative control. DNA of the human MDA-MB231 tumor cell line (MDA_w) was used as positive control.
