# Supplementary material for: Targeting TRAF3IP2, Compared to Rab27, is More Effective in Suppressing the Development and Metastasis of Breast Cancer
Source: Sci Rep. 2020 Jun 1;10:8834. doi: 10.1038/s41598-020-64781-z (PMC7264196; doi:10.1038/s41598-020-64781-z)
Supplement: Supplementary file 2 — Supplementary Tables. [file 41598_2020_64781_MOESM2_ESM.pdf]

Supplementary Tables

Supplementary Table 1				
**Gene**	**Comparison**	**Mean Difference ± Standard Error of diff.**	**Confidence interval**	**P-value**
CDH2				
	MDA_{w} vs. $MDA_{KDRab27a}$	-25.81 ± 1.692	-31.00 to -20.61	<0.0001
	MDA_{w} vs. $MDA_{KDTRAF3IP2}$	-23.75 ± 1.692	-28.94 to -18.56	<0.0001
	$MDA_{KDRab27a}$ vs. $MDA_{KDTRAF3IP2}$	2.057 ± 1.692	-3.135 to 7.249	ns
MCAM				
	MDA_{w} vs. $MDA_{KDRab27a}$	6.006 ± 1.764	0.5954 to 11.42	<0.05
	MDA_{w} vs. $MDA_{KDTRAF3IP2}$	-6.012 ± 1.764	-11.43 to -0.5982	<0.05
	$MDA_{KDRab27a}$ vs. $MDA_{KDTRAF3IP2}$	-12.02 ± 1.764	-17.43 to -6.607	<0.01
SERPINB5				
	MDA_{w} vs. $MDA_{KDRab27a}$	20.77 ± 4.013	7.716 to 33.83	<0.01
	MDA_{w} vs. $MDA_{KDTRAF3IP2}$	42.80 ± 4.487	28.20 to 57.40	<0.001
	$MDA_{KDRab27a}$ vs. $MDA_{KDTRAF3IP2}$	22.02 ± 4.487	7.423 to 36.62	<0.05
ANGPT1				
	MDA_{w} vs. $MDA_{KDRab27a}$	-8.434 ± 3.131	-18.04 to 1.172	ns
	MDA_{w} vs. $MDA_{KDTRAF3IP2}$	11.78 ± 3.131	2.173 to 21.39	<0.05
	$MDA_{KDRab27a}$ vs. $MDA_{KDTRAF3IP2}$	20.21 ± 3.131	10.61 to 29.82	<0.01
ANGPTL4				
	MDA_{w} vs. $MDA_{KDRab27a}$	34.06 ± 1.226	30.30 to 37.82	<0.0001
	MDA_{w} vs. $MDA_{KDTRAF3IP2}$	35.52 ± 1.226	31.76 to 39.28	<0.0001
	$MDA_{KDRab27a}$ vs. $MDA_{KDTRAF3IP2}$	1.457 ± 1.226	-2.306 to 5.220	ns
KLRC2				
	MDA_{w} vs. $MDA_{KDRab27a}$	-8.478 ± 2.039	-14.73 to -2.223	<0.05
	MDA_{w} vs. $MDA_{KDTRAF3IP2}$	-1.985 ± 2.039	-8.240 to 4.270	ns
	$MDA_{KDRab27a}$ vs. $MDA_{KDTRAF3IP2}$	6.493 ± 2.039	0.2375 to 12.75	<0.05
ALDH3A				
	MDA_{w} vs. $MDA_{KDRab27a}$	7.414 ± 0.6819	5.322 to 9.506	<0.0001
	MDA_{w} vs. $MDA_{KDTRAF3IP2}$	6.046 ± 0.6819	3.954 to 8.138	<0.001
	$MDA_{KDRab27a}$ vs. $MDA_{KDTRAF3IP2}$	-1.368 ± 0.6819	-3.460 to 0.7242	ns
CXCL12				
	MDA_{w} vs. $MDA_{KDRab27a}$	18.06 ± 1.152	14.31 to 21.81	<0.0001
	MDA_{w} vs. $MDA_{KDTRAF3IP2}$	18.25 ± 1.031	14.90 to 21.61	<0.0001
	$MDA_{KDRab27a}$ vs. $MDA_{KDTRAF3IP2}$	0.1894 ± 1.152	-3.561 to 3.939	ns
TGFβ1				
	MDA_{w} vs. $MDA_{KDRab27a}$	-36.85 ± 6.713	-57.45 to -16.26	<0.01
	MDA_{w} vs. $MDA_{KDTRAF3IP2}$	22.82 ± 6.713	2.219 to 43.41	<0.05
	$MDA_{KDRab27a}$ vs. $MDA_{KDTRAF3IP2}$	59.67 ± 6.713	39.07 to 80.27	<0.001
MAP2K5				
	MDA_{w} vs. $MDA_{KDRab27a}$	-20.21 ± 5.656	-37.56 to -2.855	<0.05
	MDA_{w} vs. $MDA_{KDTRAF3IP2}$	7.414 ± 5.656	-9.940 to 24.77	ns
	$MDA_{KDRab27a}$ vs. $MDA_{KDTRAF3IP2}$	27.62 ± 5.656	10.27 to 44.98	<0.01
MMP7				
	MDA_{w} vs. $MDA_{KDRab27a}$	-6.439 ± 2.109	-12.91 to 0.03112	ns

	MDA_w vs. $MDA_{KDTRAF3IP2}$	8.713 ± 2.109	2.242 to 15.18	<0.05
	$MDA_{KDRab27a}$ vs. $MDA_{KDTRAF3IP2}$	15.15 ± 2.109	8.682 to 21.62	<0.001
TIMP1				
	MDA_w vs. $MDA_{KDRab27a}$	-14.77 ± 3.068	-25.70 to -3.834	<0.05
	MDA_w vs. $MDA_{KDTRAF3IP2}$	37.48 ± 3.068	26.55 to 48.42	<0.001
	$MDA_{KDRab27a}$ vs. $MDA_{KDTRAF3IP2}$	52.25 ± 3.360	40.28 to 64.23	<0.001
TIMP2				
	MDA_w vs. $MDA_{KDRab27a}$	-57.59 ± 7.873	-83.21 to -31.97	<0.01
	MDA_w vs. $MDA_{KDTRAF3IP2}$	77.02 ± 7.042	54.10 to 99.93	<0.001
	$MDA_{KDRab27a}$ vs. $MDA_{KDTRAF3IP2}$	134.6 ± 7.873	109.0 to 160.2	<0.0001

Supplementary Table 1. Gene expression levels of Rab27a and TRAF3IP2 silenced breast cancer cells compared to MDA_W. The analysis of variance method was used for each gene separately. The graph is shown in Figure 2A.

Supplementary Table 2				
**Comparison Tumor weight of experimental series I**	**Mean Difference ± Standard Error**	**Confidence interval**	**P-value**	
MDA_w 8 wks. vs. $MDA_{KDRab27a}$ 15 wks.	4266 ± 635.3	2502 to 6030	<0.01	Significantly lower tumorigenesis of $MDA_{KDRab27a}$
MDA_w 8 wks. vs. $MDA_{KDRab27a}$ 30 wks.	2834 ± 860.6	94.98 to 5572	<0.05	Significantly lower tumorigenesis of $MDA_{KDRab27a}$
MDA_w 8 wks. vs. $MDA_{KDTRAF3IP2}$ 15 wks.	4279 ± 635.3	2516 to 6043	<0.01	Significantly lower tumorigenesis of $MDA_{KDTRAF3IP2}$
MDA_w 8 wks. vs. $MDA_{KDTRAF3IP2}$ 52 wks.	4181 ± 637.4	2412 to 5951	<0.01	Significantly lower tumorigenesis of $MDA_{KDTRAF3IP2}$
$MDA_{KDRab27a}$ 15 wks. vs. $MDA_{KDTRAF3IP2}$ 15 wks.	13.03 ± 10.74	-16.77 to 42.84	ns	Lower tumorigenesis of $MDA_{KDTRAF3IP2}$
$MDA_{KDRab27a}$ 15 wks. vs. $MDA_{KDTRAF3IP2}$ 52 wks.	-98.00 ± 53.11 .	-245.5 to 49.46	ns	Lower tumorigenesis of $MDA_{KDTRAF3IP2}$
$MDA_{KDRab27a}$ 30 wks. vs. $MDA_{KDRab27a}$ 15 wks.	1433 ± 261.2	601.4 to 2264	<0.05	Slightly lower tumorigenesis of $MDA_{KDRab27a}$ at 30 wks
$MDA_{KDRab27a}$ 30 wks. vs. $MDA_{KDTRAF3IP2}$ 15 wks.	1446 ± 260.9	615.3 to 2276	<0.05	Significantly lower tumorigenesis of $MDA_{KDRab27a}$ at 15 wks
$MDA_{KDRab27a}$ 30 wks. vs. $MDA_{KDTRAF3IP2}$ 52 wks.	1348 ± 269.7	489.4 to 2206	<0.05	Significantly lower tumorigenesis of $MDA_{KDTRAF3IP2}$
$MDA_{KDTRAF3IP2}$ 15 wks. vs. $MDA_{KDTRAF3IP2}$ 52 wks.	-84.97 ± 53.84	-234.4 to 64.51	ns	Non significant tumorigenesis of $MDA_{KDTRAF3IP2}$ between 15-52 wks

Supplementary Table 2. Detailed statistical results of the tumor weight of the experimental series I. To analyze to possible existence of significant differences between the tumor weight of MDA_w, $MDA_{KDRab27a}$ and $MDA_{KDTRAF3IP2}$ we used analysis of variance method to compare each group after 8, respectively 15, 30 or 52 weeks with each other. We saw that MDA_w after 8 weeks was significantly bigger in comparison to $MDA_{KDRab27a}$ or $MDA_{KDTRAF3IP2}$ at any time point. $MDA_{KDRab27a}$ after 30 weeks was significantly bigger than $MDA_{KDRab27a}$ after 15 weeks or $MDA_{KDTRAF3IP2}$ after 15 or 52 weeks. No significant difference was found between $MDA_{KDRab27a}$ after 15 weeks vs. $MDA_{KDTRAF3IP2}$ after 15 or 52 weeks or between $MDA_{KDTRAF3IP2}$ after 15 and 52 weeks. The graph is shown in Figure 4.

Supplementary Table 3			
**Comparison Tumor weight of experimental series II**	**Mean Difference ± Standard Error of diff.**	**Confidence interval**	**P-value**
MSCs coinjected with MDA_w 8 wks. vs. MDA_w 8 wks.	4258 ± 371.7	5290 to 3226	<0.001
MSCs coinjected with MDA_w 8 wks. vs. MSCs coinjected with $MDA_{KDRab27a}$ 15 wks.	65.00 ± 26.44	-8.4 to 138.4	ns
MSCs coinjected with MDA_w 8 wks. vs. $MDA_{KDRab27a}$ 15 wks.	57.10 ± 26.56	-16.65 to 130.8	ns
MSCs coinjected with MDA_w 8 wks. vs. MSCs coinjected with $MDA_{KDRab27a}$ 30 wks.	62.33 ± 26.55	-11.38 to 136.0	ns
MSCs coinjected with MDA_w 8 wks. vs. $MDA_{KDRab27a}$ 30 wks.	-1298 ± 437.8	- 2514 to - 82.35	<0.01
MSCs coinjected with MDA_w 8 wks. vs. MSCs coinjected with $MDA_{KDTRAF3IP2}$ 15 wks.	68.67 ± 26.36	-4.517 to 141.8	ns
MSCs coinjected with MDA_w 8 wks. vs. $MDA_{KDTRAF3IP2}$ 15 wks.	76.47 ± 17.88	-26.82 to 126.1	ns
MSCs coinjected with MDA_w 8 wks. vs. MSCs coinjected with $MDA_{KDTRAF3IP2}$ 52 wks.	78.67 ± 17.84	-29.15 to 128.2	ns
MSCs coinjected with MDA_w 8 wks. vs. $MDA_{KDTRAF3IP2}$ 52 wks.	85.37 ± 35.13	-29.15 to 128.2	ns
MDA_w 8 wks. vs. MSCs coinjected with $MDA_{KDRab27a}$ 15 wks.	4323 ± 370.8	3293 to 5352	<0.001
MDA_w 8 wks. vs. $MDA_{KDRab27a}$ 15 wks.	4315 ± 370.8	3285 to 5344	<0.001
MDA_w 8 wks. vs. MSC coinjected with $MDA_{KDRab27a}$ 30 wks.	4320 ± 370.8	3290 to 5350	<0.001
MDA_w 8 wks. vs. $MDA_{KDRab27a}$ 30 wks.	2960 ± 573.2	1368 to 4551	<0.01
MDA_w 8 wks. vs. MSC coinjected with $MDA_{KDTRAF3IP2}$ 15 wks.	4326 ± 370.8	3297 to 5356	<0.001
MDA_w 8 wks. vs. $MDA_{KDTRAF3IP2}$ 15 wks.	4324 ± 370.8	3295 to 5354	<0.001
MDA_w 8 wks. vs. MSC coinjected with $MDA_{KDTRAF3IP2}$ 52 wks.	4326 ± 370.8	-3297 to 5356	<0.001
MDA_w 8 wks. vs. $MDA_{KDTRAF3IP2}$ 52 wks.	4326 ± 370.8	3297 to 5356	<0.001
MSCs coinjected with $MDA_{KDRab27a}$ 15 wks. vs. $MDA_{KDRab27a}$ 15 wks.	-7.900 ± 3.853	-18.60 to 2.799	ns
MSCs coinjected with $MDA_{KDRab27a}$ 15 wks. vs. MSCs coinjected with $MDA_{KDRab27a}$ 30 wks.	-2.667 ± 3.771	-13.14 to 7.804	ns

MSCs coinjected with MDA$_{KDRab27a}$ 15 wks. vs. MDA$_{KDRab27a}$ 30 wks.	-1363 ± 437.1	2576 to 149.5	<0.05
MSCs coinjected with MDA$_{KDRab27a}$ 15 wks. vs. MSCs coinjected with MDA$_{KDTRAF3IP2}$ 15 wks.	3.667 ± 2.028	-1.963 to 9.296	ns
MSCs coinjected with MDA$_{KDRab27a}$ 15 wks. vs. MSCs coinjected with MDA$_{KDTRAF3IP2}$ 15 wks.	1.467 ± 2.409	-5.221 to 8.154	ns
MSCs coinjected with MDA$_{KDRab27a}$ 15 wks. vs. MSCs coinjected with MDA$_{KDTRAF3IP2}$ 52 wks.	3.667 ± 2.028	-1.963 to 9.296	ns
MSCs coinjected with MDA$_{KDRab27a}$ 15 wks. vs. MDA$_{KDTRAF3IP2}$ 52 wks.	3.667 ± 2.028	-1.963 to 9.296	ns
MDA$_{KDRab27a}$ 15 wks. vs. MSCs coinjected with MDA$_{KDRab27a}$ 30 wks.	5.233 ± 4.566	-7.444 to 17.91	ns
MDA$_{KDRab27a}$ 15 wks. vs. MDA$_{KDRab27a}$ 30 wks.	-1355 ± 437.1	-2569 to -141.6	<0.05
MDA$_{KDRab27a}$ 15 wks. vs. MSCs coinjected with MDA$_{KDTRAF3IP2}$ 15 wks.	11.57 ± 3.277	2.469 to 20.66	<0.05
MDA$_{KDRab27a}$ 15 wks. vs. MDA$_{KDTRAF3IP2}$ 15 wks.	9.367 ± 3.525	-0.4212 to 19.15	ns
MDA$_{KDRab27a}$ 15 wks. vs. MSCs coinjected with MDA$_{KDTRAF3IP2}$ 52 wks.	11.57 ± 3.277	2.469 to 20.66	<0.05
MDA$_{KDRab27a}$ 15 wks. vs. MDA$_{KDTRAF3IP2}$ 52 wks.	11.57 ± 3.277	2.469 to 20.66	<0.05
MSCs coinjected with MDA$_{KDRab27a}$ 30 wks. vs. MDA$_{KDRab27a}$ 30 wks.	-1360 ± 437.1	-2574 to -146.9	<0.05
MSCs coinjected with MDA$_{KDRab27a}$ 30 wks. vs. MSCs coinjected with MDA$_{KDTRAF3IP2}$ 15 wks.	6.333 ± 3.180	-2.495 to 15.16	ns
MSCs coinjected with MDA$_{KDRab27a}$ 30 wks. vs. MDA$_{KDTRAF3IP2}$ 15 wks.	4.133 ± 3.435	-5.405 to 13.67	ns
MSCs coinjected with MDA$_{KDRab27a}$ 30 wks. vs. MSCs coinjected with MDA$_{KDTRAF3IP2}$ 52 wks.	6.333 ± 3.180	-2.495 to 15.16	ns
MSCs coinjected with MDA$_{KDRab27a}$ 30 wks. vs. MDA$_{KDTRAF3IP2}$ 52 wks.	6.333 ± 3.180	-2.495 to 15.16	ns
MDA$_{KDRab27a}$ 30 wks. vs. MSCs coinjected with MDA$_{KDTRAF3IP2}$ 15 wks.	1367 ± 437.0	153.2 to 2580	<0.05
MDA$_{KDRab27a}$ 30 wks. vs. MDA$_{KDTraf3IP2}$ 15 wks.	1364 ± 437.1	151.0 to 2578	<0.05
MDA$_{KDRab27a}$ 30 wks. vs. MSCs coinjected with MDA$_{KDTRAF3IP2}$ 52 wks.	1367 ± 437.0	153.2 to 2580	<0.05
MDA$_{KDRab27a}$ 30 wks. vs. MDA$_{KDTRAF3IP2}$ 52 wks.	1367 ± 437.0	153.2 to 2580	<0.05

$MDA_{KDTRAF3IP2}$ 15 wks. vs. MSCs coinjected with $MDA_{KDTRAF3IP2}$ 15 wks.	2.200 ± 1.300	-1.409 to 5.809	ns
$MDA_{KDTRAF3IP2}$ 15 wks. vs. MSCs coinjected with $MDA_{KDTRAF3IP2}$ 52 wks.	2.200 ± 1.300	-1.409 to 5.809	ns
$MDA_{KDTRAF3IP2}$ 15 wks. vs. $MDA_{KDTraf3IP2}$ 52 wks.	2.200 ± 1.300	-1.409 to 5.809	ns

Supplementary Table 3. Detailed statistical results of the tumor weight of the experimental series II. To analyze to possible existence of significant differences between the tumor weight of MDA_w, $MDA_{KDRab27a}$ and $MDA_{KDTRAF3IP2}$ coinjected with naïve MSCs we used analysis of variance method to compare each group after 8, respectively 15, 30 or 52 weeks with each other. We saw that tumors created by MDAw after 8 weeks were significantly heavier in comparison to $MDA_{KDRab27a}$, $MDA_{KDTRAF3IP2}$ or any tumor-like masses produced by MSCs at any time point. $MDA_{KDRab27a}$ after 30 weeks was significantly heavier than any other tumor or tumor-like mass except MDA_w. No tumor growth was seen in $MDA_{KDTRAF3IP2}$ after 52 weeks as well as MSC coinjected with $MDA_{KDTRAF3IP2}$ after 15 or 52 weeks. The graph is shown in Figure 5.
